# Pectin Metabolism Influences Phloem Architecture and Flowering Time in *Arabidopsis Thaliana*

**DOI:** 10.1002/advs.202502980

**Published:** 2025-07-29

**Authors:** Qing Zhang, Juan Du, Shuang Hao, Zhengmei Leng, Weiguo Liu, Chengjian Zhao, Yue Yu, Longquan Chen, Charles T. Anderson, Chaowen Xiao

**Affiliations:** ^1^ Key Laboratory of Bio‐Resource and Eco‐Environment of Ministry of Education, College of Life Sciences Sichuan University Chengdu 610064 China; ^2^ College of Agronomy Sichuan Agricultural University Chengdu 611130 China; ^3^ State Key Laboratory of Biotherapy and Cancer Center, West China Hospital Sichuan University and Collaborative Innovation Center Chengdu 610041 China; ^4^ School of Physics University of Electronic Science and Technology of China Chengdu 611731 China; ^5^ Department of Biology, The Pennsylvania State University University Park PA 16802 USA

**Keywords:** auxin, cell wall, flowering time, FT, homogalacturonan, pectin modification, phloem, wall mechanics

## Abstract

Cell wall matrices undergo continuous remodeling during plant growth and development. The methyl‐ and de‐methyl‐esterification of pectic homogalacturonan dynamically influence the physicochemical and mechanical properties of cell walls, and consequently regulate organ morphogenesis. Phloem plays a crucial role in the transport of photoassimilates and other organic molecules from source to sink tissues, and the walls of phloem cells are rich in pectins. However, how pectin metabolism orchestrates phloem architecture and function is unclear. This work demonstrates that the methyl‐esterification status and subsequent degradation of pectic homogalacturonan affect flowering time by modulating phloem development and transport capacity, which control the transport of a mobile florigen, FLOWERING LOCUS T protein, in the phloem stream. During this process, auxin signaling is stimulated by pectin de‐methyl‐esterification to influence vasculogenesis. Together, this data provide new insights into the mechanisms by which pectin chemistry is coordinated with auxin signaling to mediate phloem development, long‐distance transport and flowering time.

## Graphical abstract

Cell wall remodeling mediated by changes in pectic homogalacturonan, including its methyl‐esterification status and degradation, shapes wall architecture. This study finds that pectin metabolism dynamically modulates wall properties and triggers auxin responses, which influence phloem morphogenesis and function, mediating the long‐distance transport of the mobile florigen, FLOWERING LOCUS T, in the phloem stream and regulating flowering time in *Arabidopsis thaliana*.

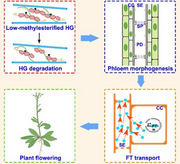

## 1 Introduction

The outermost layer of growing plant cells is the primary cell wall, which is constituted of multiple polysaccharide polymers including cellulose, hemicelluloses and pectins, as well as small amounts of structural proteins.^[^ ^1^, ^2^ ^]^ The composition and physical properties of cell walls determine cell expansion, cell shape and organ morphogenesis.^[^ ^3^, ^4^, ^5^ ^]^ Pectins, a class of polysaccharides enriched in galacturonic acid (GalA), are mainly composed of homogalacturonan (HG), rhamnogalacturonan (RG) I and RG II. They serve important functions in wall assembly and plant development.^[^ ^6^, ^7^ ^]^ HG is an unbranched homopolymer of α‐1,4‐linked D‐GalA that is synthesized in the Golgi apparatus and secreted into the apoplast in a highly methyl‐esterified form, and is subsequently de‐esterified by pectin methyl‐esterases (PMEs).^[^ ^8^, ^9^, ^10^ ^]^ In the cell wall, pectin methyl‐esterase inhibitors (PMEIs) antagonize the de‐methyl‐esterification process.^[^ ^11^ ^]^ De‐methyl‐esterified HGs can either be prone to be targets of pectin‐degrading enzymes such as polygalacturonases (PGs) to loosen the wall, or be crosslinked by calcium to strengthen the wall.^[^ ^6^ ^]^ The processivity of different PMEs influences these outcomes, since non‐processive PMEs would be expected to generate sporadically de‐methyl‐esterified HG, whereas processive PMEs produce stretches of de‐methyl‐esterified HG that can undergo calcium‐mediated crosslinking. The effect of PME activity is implicated in wall porosity, elasticity, pH and ionic status, as well as the release of important signaling molecules such as methanol and oligogalacturonides.^[^ ^12^, ^13^ ^]^ Alterations in HG methyl‐esterification levels influence a variety of physiological processes, such as fertilization,^[^ ^14^, ^15^ ^]^ stomatal movement,^[^ ^16^, ^17^ ^]^ organ initiation,^[^ ^18^, ^19^, ^20^ ^]^ pavement cell morphogenesis,^[^ ^21^, ^22^ ^]^ root development,^[^ ^23^ ^]^ seed mucilage extrusion,^[^ ^24^ ^]^ seed germination,^[^ ^25^ ^]^ and response to external stimuli.^[^ ^26^ ^]^ However, it is unclear how pectin metabolism regulates vascular formation and plant flowering.

Flowering is the deterministic transition from a vegetative to a reproductive stage. The success of reproductive growth depends on flowering, which consequently affects crop yields. FLOWERING LOCUS T (FT) integrates different signaling pathways and acts as a critical nexus in the control of flowering time.^[^ ^27^, ^28^ ^]^ FT protein is specifically synthesized in phloem companion cells of leaf veins and transported through the phloem to the shoot apical meristem (SAM) to induce flowering.^[^ ^29^ ^]^ The phloem of vascular plants contains an effective microfluidics system, including sieve elements (SEs), companion cells (CCs) and plasmodesmata (PD), which enables the selective and efficient transport of sugars and nutrients, proteins, and mRNAs over long distances.^[^ ^30^ ^]^ The velocity and volume of transport by the phloem depend essentially on the pressure gradient between source and sink, but the geometry of the microfluidic sieve tube, especially the anatomy of sieve tubes and sieve plate pores, also has profound impacts on these parameters.^[^ ^31^ ^]^ Phloem cells are rich in wall polysaccharides, and cell walls of mature SEs contain a dense inner pectin‐rich layer^[^ ^32^ ^]^ that has been proposed to influence the mechanical properties of sieve tubes.^[^ ^33^ ^]^ Additionally, pectin‐modifying enzymes have been implicated in the control of flowering time^[^ ^34^ ^]^ and phloem transport,^[^ ^35^ ^]^ and have been detected in the PD proteome from walls of Arabidopsis suspension cells.^[^ ^36^, ^37^ ^]^ However, a comprehensive view of how wall modifications affect phloem architecture and function is still lacking.

The developmental patterning of vascular tissue is a prerequisite for its function and involves multiple regulatory mechanisms.^[^ ^38^ ^]^ Among these regulatory factors, auxin signaling plays a central role in vascular tissue specification, and this process is mediated by auxin response factors.^[^ ^39^ ^]^ The polarity of the auxin efflux carrier PIN‐FORMED1 (PIN1) defines the direction of auxin flow and the position of newly emerging leaf vasculature.^[^ ^40^, ^41^ ^]^ The physical status of the cell wall can also have profound impacts on auxin distribution and accumulation.^[^ ^42^, ^43^, ^44^ ^]^ Specifically, it has been demonstrated that PIN1 localization responds to wall mechanical stresses.^[^ ^45^ ^]^ Thus, cell wall remodeling and resultant mechanical changes can act as initiating signals to trigger auxin responses and tissue growth.

In this study, we found that variations in pectic HG methyl‐esterification mediated by alterations in the expression of *PME5*, *PMEI1* and *POLYGALACTURONASE INVOLVED IN EXPANSION2* (*PGX2*) affect plant flowering time, which correlates with the transport efficiency of mobile FT protein in the phloem. Through in‐depth dissection of the phloem system including sieve elements, sieve pores and plasmodesmata, we discovered changes in phloem development and architecture in response to these genetic changes, which led to the alteration of long‐distance transport capacity of the phloem. Importantly, changes in pectic HG chemistry act as an initiating signal to trigger altered wall mechanics and auxin signaling pathways. These results emphasize the role of dynamic pectin modifications in phloem architecture establishment and plant developmental regulation, highlighting the inherent connections between wall chemistry, wall mechanics, hormone signaling, and organ morphogenesis.

## 2 Results

### 2.1 Alterations in Pectic HG Methyl‐Esterification Status Affect Flowering Time

**Figure 1:**
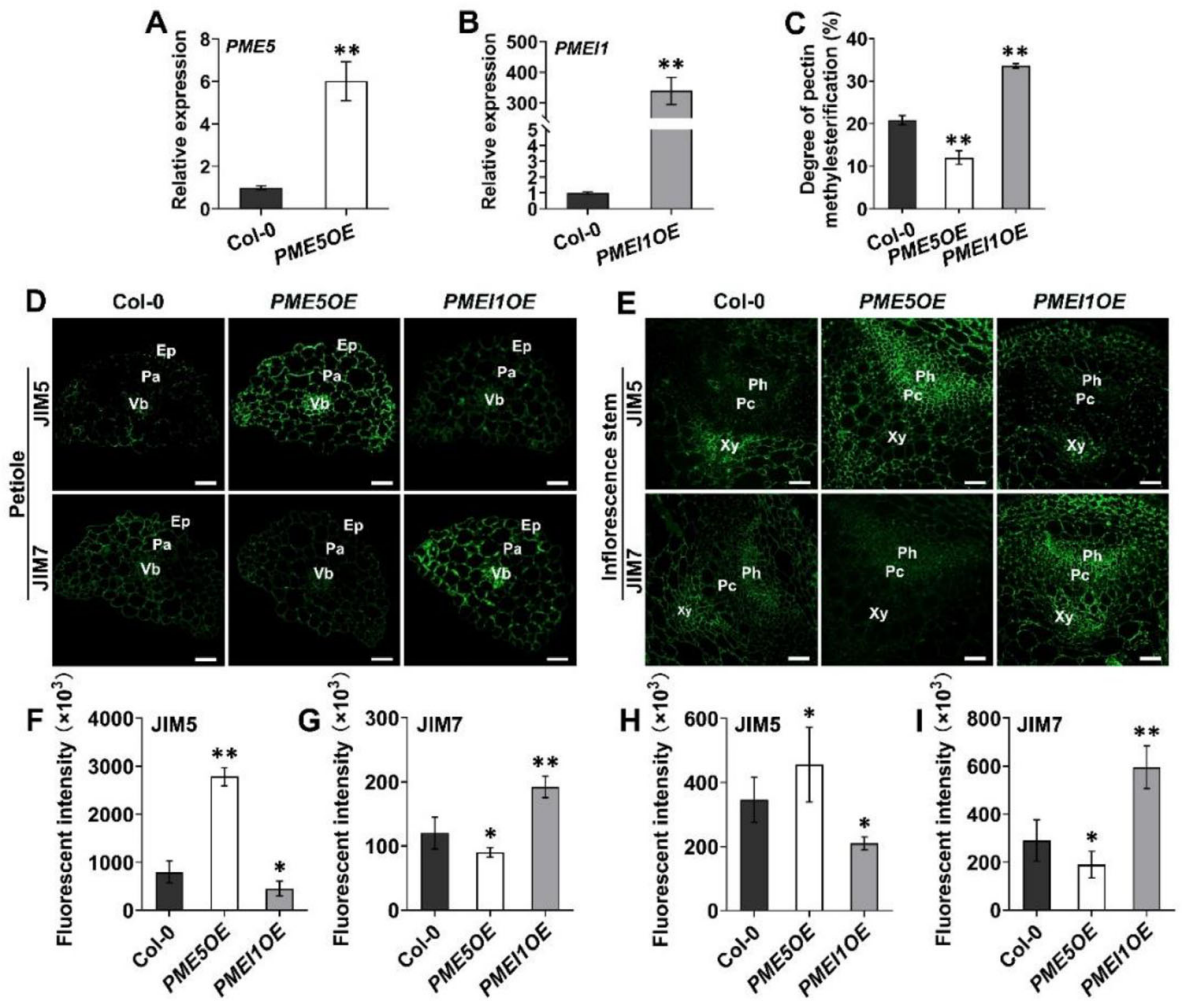
PME5 and PMEI1 mediate methyl‐esterification status of pectic HG. A,B) Gene expression detection of *PME5* and *PMEI1* in 6‐day‐old light‐grown Col‐0, *PME5OE* and *PMEI1OE* seedlings by RT‐qPCR (n = 3). *ACT2* was defined as a reference gene. Data are representative of two independent experiments. C) The profiling of HG methyl‐esterification degree in 6‐day‐old light‐grown seedlings of Col‐0, *PME5OE* and *PMEI1OE* (n = 5). Data are representative of three independent experiments. D,E) Immunolabelling of low‐methyl‐esterified HG (JIM5) and high‐methyl‐esterified HG (JIM7) on the cross sections of 11‐day‐old Col‐0, *PME5OE* and *PMEI1OE* petioles (D) and 35‐day‐old Col‐0, *PME5OE* and *PMEI1OE* inflorescence stems (E). Ep, epidermis; Pa, parenchyma; Vb, vascular bundle; Ph, phloem; Pc, procambium; Xy, xylem. Scale bar = 40 µm. F–I) Fluorescent intensity of immunolabelling images from petioles (F and G) and stems (H and I) (n ≥ 6 images from at least 3 seedlings per genotype. Data represent three independent experiments). Error bars represent SD. *p < 0.05, **p < 0.001, Student's *t*‐test.

**Figure 2:**
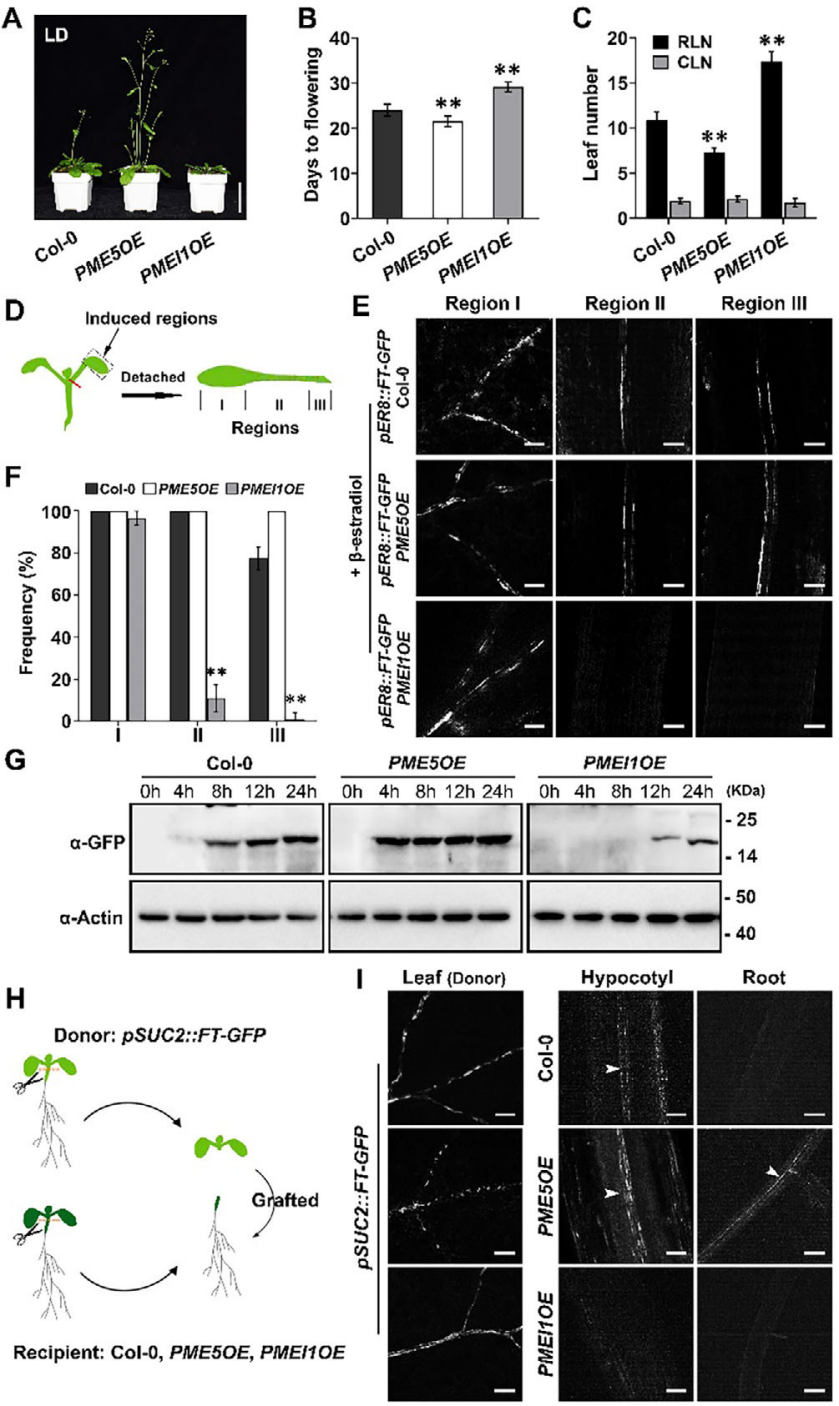
HG de‐methylesterification in cell wall promotes flowering and FT protein transport. A) 30‐day‐old Col‐0, *PME5OE* and *PMEI1OE* plants grown in long‐day (LD) conditions. Scale bar = 5 cm. B) Days to flowering of Col‐0, *PME5OE* and *PMEI1OE* plants in LD conditions (n ≥ 30 plants per genotype. Data are representative of three independent experiments). C) Numbers of rosette leaves (RLN) and cauline leaves (CLN) of Col‐0, *PME5OE* and *PMEI1OE* plants in LD conditions (n ≥ 30 plants per genotype). Data are representative of three independent experiments. D) Schematic illustration for β‐estradiol induction and observation of FT‐GFP in the cotyledons. Dashed rectangle marks the region for β‐estradiol induction. The detached leaves were divided into region I (leaf part), II (three‐quarters of petiole close to leaf), and III (one‐quarter of petiole close to shoot). E) Confocal images of FT‐GFP distribution after 20 µM β‐estradiol treatment on the leaf regions of 11‐day‐old *pER8::FT‐GFP* transgenic plants. Scale bar = 100 µm. F) Frequency of FT‐GFP signal in different regions of induced leaves. Error bars represent SD (n ≥ 50 images from at least 30 seedlings. The data are the sum of three independent experiments). G) Time‐course detection of FT‐GFP protein in the shoot apices after plants have bolted for 5 cm. Samples were collected at 0, 4, 8, 12, and 24 h after β‐estradiol induction in *pER8::FT‐GFP* transgenic plants under different backgrounds. The shoot apices from 30 plants for each genotype were used for western blot. H) Illustration of a hypocotyl‐grafting procedure. Upper hypocotyl part close to shoot was cut from both donor and recipient plants as depicted. The donor shoot (*pSUC2::FT‐GFP*) was then transferred to a recipient plant (Col‐0, *PME5OE* and *PMEI1OE*) individually. I) Detection of FT‐GFP signal in the cotyledon vein of *pSUC2::FT‐GFP* donor, and the vasculature of hypocotyls and roots of recipient (Col‐0, *PME5OE* and *PMEI1OE*). At least 10 plants per genotype were analyzed in one replicate. Scale bar = 100 µm. Error bars represent SD. **p < 0.001, Student's *t*‐test.

To explore the role of pectic HG with varying methyl‐esterification status in plant development, *PME5* and *PMEI1*, which have been characterized for enzyme activity and functional relevance to a variety of biological processes,^[^ ^18^, ^19^, ^21^, ^46^ ^]^ were chosen for this study. We obtained Arabidopsis seeds of *PMEI1OE* from Dr. Vincenzo Lionetti,^[^ ^46^ ^]^ and in parallel generated *PME5* overexpressing transgenic lines (*PME5OE*). The increased expression levels of *PME5* and *PMEI1* were verified by RT‐qPCR (**Figure** **1**A,B). Biochemical measurements showed that the degree of HG methyl‐esterification was lower in *PME5OE* seedlings, whereas it was higher in *PMEI1OE* seedlings, compared with Col‐0 controls (Figure 1C). To visualize cell wall modifications that occurred as results of the overexpression of *PME5* and *PMEI1*, immunolabeling experiments were performed in petioles and inflorescence stems that exhibit rapid growth and yield clear immunolabeling results. Compared with Col‐0 and *PMEI1OE* plants, labeling intensity with JIM5, which binds low‐methyl‐esterified HG,^[^ ^47^, ^48^ ^]^ was higher in the epidermis, parenchyma, and vascular bundles of *PME5OE* petioles. In contrast, the labeling intensity of JIM7, which binds high‐methyl‐esterified HG,^[^ ^47^, ^48^ ^]^ was the strongest in petioles of *PMEI1OE* compared with Col‐0 and *PME5OE* plants (Figure 1D,F,G). The same phenomenon was observed in inflorescence stems (Figure 1E,H,I). These results indicate that overexpression of *PME5* and *PMEI1* decreased and increased pectic HG methyl‐esterification, respectively, in the wall. Notably, we observed that *PME5OE* plants bolted earlier under long‐day (LD) conditions, whereas *PMEI1OE* plants bolted later than Col‐0 controls (**Figure** **2**A–C). Considering the central role of FT in flowering onset and the characteristic of FT as a phloem‐mobile signal molecule,^[^ ^29^ ^]^ we next investigated whether cell wall modification mediates FT protein transport in the phloem.

### 2.2 De‐Methyl‐Esterification of Pectic HG in the Cell Wall Accelerates FT Protein Transport

To explore the impact of cell wall modification on flowering time and FT protein transport, we expressed β‐estradiol‐inducible FT‐GFP in Col‐0, *PME5OE* and *PMEI1OE* plants individually to monitor FT protein trafficking. As illustrated in Figure 2D, the leaf with blade and petiole was divided into three regions, which were subjected to microscopic examination and calculation of the frequency of fluorescent signal in these regions.^[^ ^49^ ^]^ In leaves of *pER8::FT‐GFP* Col‐0 seedlings, the GFP signal was observed in vascular tissue of the three regions after β‐estradiol application, but not in untreated leaves (Figure S1A, Supporting Information). When β‐estradiol was continuously applied to *pER8::FT‐GFP* Col‐0 seedlings, plants flowered earlier (Figure S1B,C, Supporting Information), confirming that the *pER8::FT‐GFP* construct is fully functional. 3 days after β‐estradiol treatment of *pER8::FT‐GFP* transgenic plants, we found the fluorescent signal of FT‐GFP was comparable in region I of Col‐0, *PME5OE*, and *PMEI1OE* plants (Figure 2E,F). In the Col‐0 background, GFP signal was present at a frequency of 100% in region II, and ≈70% in region III. In *pER8::FT‐GFP PME5OE* plants, the detection frequency of GFP signal in regions I and II was similar to that in Col‐0, while the detection frequency (100%) in region III was higher than that in Col‐0. In contrast, less than 10% of collected images showed GFP signal in region II, and few signals were observed in region III of *pER8::FT‐GFP PMEI1OE* plants (Figure 2E,F). Consistent with the distribution of FT‐GFP signal, the effect of FT‐GFP in promoting flowering was compromised in *pER8::FT‐GFP PMEI1OE* plants, which still flowered late (Figure S1B,C, Supporting Information). We next carried out local FT induction in leaves and examined the accumulation of FT‐GFP proteins in shoot apex within 24 h. FT‐GFP proteins were detected after induction for 8 h in *pER8::FT‐GFP* Col‐0 plants, and proteins were detected after induction for 4 h in *pER8::FT‐GFP PME5OE* plants. However, FT‐GFP proteins in *pER8::FT‐GFP PMEI1OE* apical meristems were not detected until 12 h after induction, and protein abundance was relatively low (Figure 2G).

To further test the effect of HG methyl‐esterification levels on FT long‐distance transport, we performed a stem micrografting experiment. Considering that *FT* mRNA is expressed in phloem companion cells and the endogenous *FT* promoter is weak, *FT‐GFP* driven by the phloem companion cell‐specific promoter of *SUCROSE TRANSPORTER 2* (*SUC2*) was used to track FT protein transport in the plant.^[^ ^29^ ^]^ The aerial parts of *pSUC2::FT‐GFP* Col‐0 seedlings (donors) were grafted onto the lower parts of Col‐0, *PME5OE* and *PMEI1OE* seedlings (recipients), respectively (Figure 2H). The donor *pSUC2::FT‐GFP* Col‐0 expressed a considerable amount of FT‐GFP in leaves as observed by confocal microscopy (Figure 2I). GFP signal was detected in the vasculature of hypocotyls below the grafted junction, but not the roots, in the Col‐0 recipients. Fluorescent signal was detected in both hypocotyls and roots of the *PME5OE* recipients, but was barely observed in the *PMEI1OE* recipients (Figure 2I).

To exclude the possibility that the late flowering of *PMEI1OE* plants is due to the failure of flowering machinery in the meristem, we checked the flowering time of *PMEI1OE* plants after directly introducing FT protein to the meristem. To accomplish this, the promoter of *KNOTTED‐LIKE FROM ARABIDOPSIS THALIANA1* (*KNAT1*), specifically active in the shoot apical meristem,^[^ ^50^ ^]^ was used to drive *FT* expression in the meristem. Introducing *pKNAT1::FT‐GUS* into Col‐0 and *PMEI1OE* plants caused early flowering (Figure S2, Supporting Information). Taken together, the above experiments reveal that alterations in HG methyl‐esterification status affect flowering time by modulating the long‐distance transport of FT in the phloem.

### 2.3 HG Methyl‐Esterification Homeostasis Is Associated with Phloem Development

**Figure 3:**
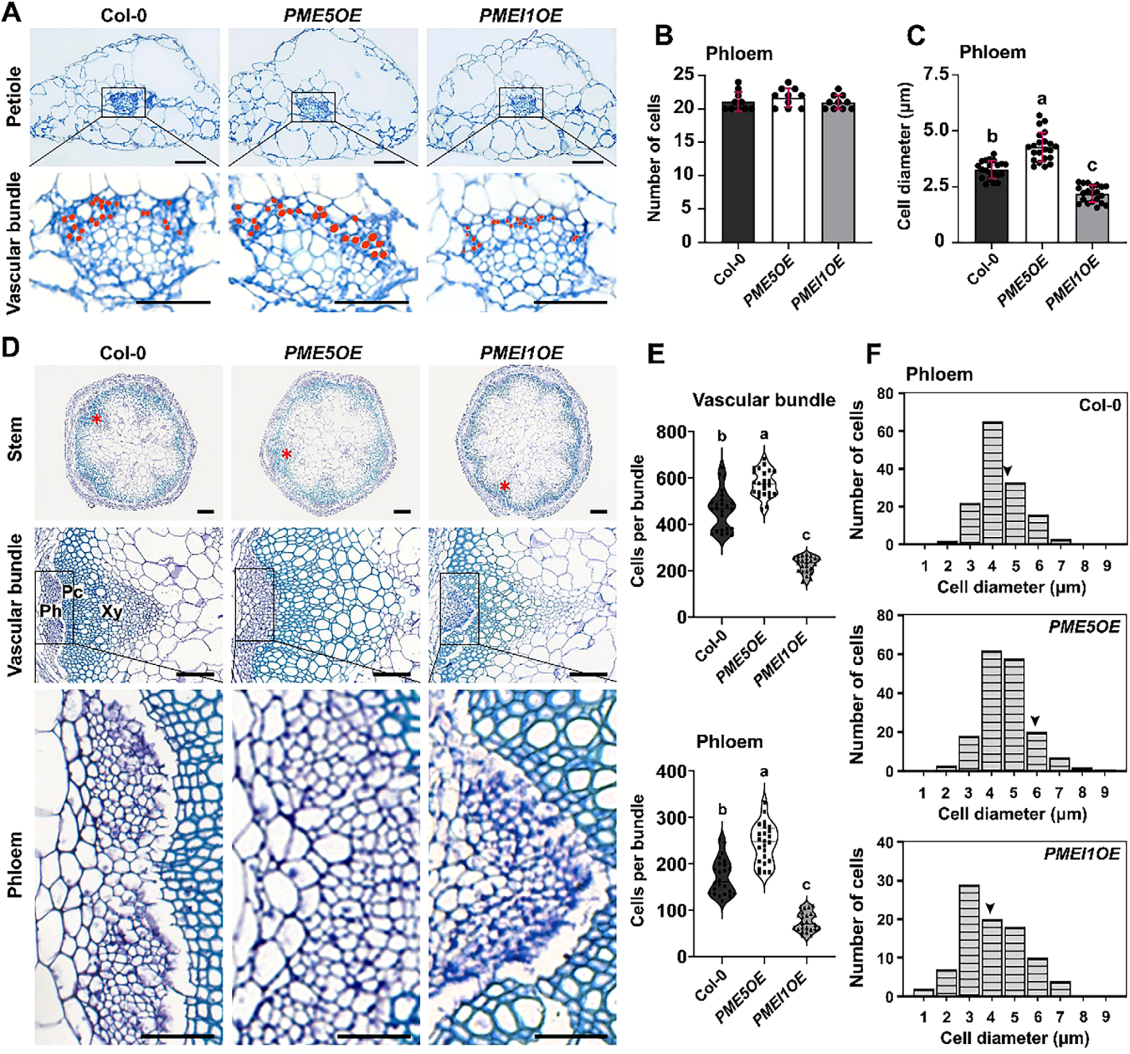
Pectin HG methyl‐esterification influences phloem development. A) Transverse sections of petioles in 11‐day‐old Col‐0, *PME5OE* and *PMEI1OE* seedlings. Transverse sections were stained with toluidine blue at 0.05% (w/v). Enlarged images of black boxes are shown in the lower row. Red dots indicate phloem cells in petioles. Scale bar = 100 µm for petiole, 50 µm for vascular bundle. B,C) The number (B) and diameter (C) of phloem cells per vascular bundle of petioles in 11‐day‐old Col‐0, *PME5OE* and *PMEI1OE* seedlings (n ≥ 10 vascular bundles for each genotype from at least 10 seedlings. Data are the sum of two independent experiments). D) Transverse sections of basal inflorescence stems in 35‐day‐old Col‐0, *PME5OE* and *PMEI1OE* plants. Close‐up images of the vascular bundles marked by red asterisks and phloem regions indicated by black boxes are shown in middle and lower row, respectively. Ph, phloem; Pc, procambium; Xy, xylem. Scale bar = 200 µm (upper), 100 µm (middle), 50 µm (lower). E) Violin plots show the cell numbers of whole vascular bundle (upper) and phloem (lower) (n ≥ 20 vascular bundles for each genotype from at least 5 inflorescence stems. Data are representative of three independent experiments). F) Distribution of the diameters of phloem cells in inflorescence stems from 35‐day‐old Col‐0, *PME5OE* and *PMEI1OE* plants (Col‐0, n = 141; *PME5OE*, n = 171; *PMEI1OE*, n = 91). Arrowheads show the average diameter of phloem cells. Data are representative of three independent experiments. Error bars represent SD. Lowercase letters indicate significantly different groups as determined by one‐way ANOVA with post‐hoc Tukey's test (*p* < 0.05).

Given that FT protein is transported in the phloem,^[^ ^29^ ^]^ and the differences in immunolabelling representing HG methyl‐esterification status in the vascular bundles of *PME5OE* and *PMEI1OE* plants, we next investigated phloem morphology in Col‐0, *PME5OE* and *PMEI1OE* plants (**Figure** **3**A). Although the number of phloem cells in petioles of *PME5OE* and *PMEI1OE* plants was the same as in Col‐0, phloem cells in *PME5OE* petioles were larger, whereas they were smaller in *PMEI1OE* petioles (Figure 3B,C). We further performed anatomical analysis of vascular structure in basal inflorescence stems of adult plants (Figure 3D). Cell number in vascular bundles encompassing phloem, procambium, and xylem was quantified. In *PME5OE* plants, total cell number was significantly higher than that in Col‐0 controls, whereas it was lower in *PMEI1OE* plants. Notably, there were the most phloem cells in *PME5OE*, and the fewest phloem cells in *PMEI1OE* stems (Figure 3E). The diameters of phloem cells also differed between the three genotypes. Cell diameter averaged 4–6 µm in *PME5OE*, 3–5 µm in Col‐0, and 3–4 µm in *PMEI1OE* (Figure 3F). These results demonstrate that the methyl‐esterification status of pectic HG influences vascular development, especially phloem formation.

### 2.4 HG Methyl‐Esterification Status Impacts the Formation of Sieve Elements and Sieve Pores

**Figure 4:**
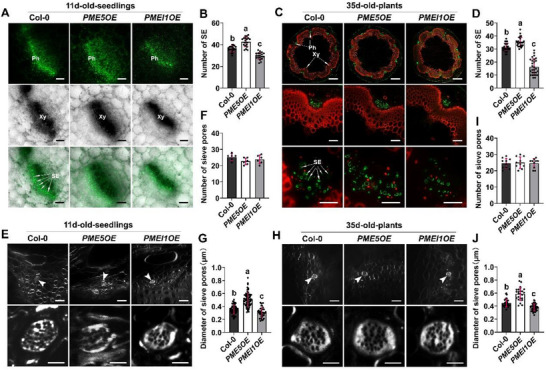
Dynamics of HG methyl‐esterification mediates the architecture of sieve elements, sieve plates in phloem. A) Immunolabelling of phloem sieve elements in the petiole midribs of 11‐day‐old Col‐0, *PME5OE* and *PMEI1OE* plants against SE‐ENOD antibody. Green signal, SE‐ENOD immunostaining. Ph, phloem; Xy, xylem; SE, sieve element. Scale bar = 50 µm. B) SE cell numbers in the petiole midribs of 11‐day‐old Col‐0, *PME5OE* and *PMEI1OE* plants described in (A) (n ≥ 20 petioles for each genotype. Data are representative of three biological replicates). C) Immunolabelling in vascular bundles of 35‐day‐old Col‐0, *PME5OE* and *PMEI1OE* stem sections. Green signal, SE‐ENOD immunostaining. Red signal, PI staining. Scale bar = 200 µm (upper), 50 µm (middle), 20 µm (lower). D) SE cell numbers in the stems of 35‐day‐old Col‐0, *PME5OE* and *PMEI1OE* plants described in (C) (n ≥ 30 vascular bundles from at least 5 stems for each genotype). E,H) Cross sections of the petioles of 11‐day‐old (E) and the stems of 35‐day‐old (H) Col‐0, *PME5OE* and *PMEI1OE* plants. Sieve plates are indicated by white arrowheads. Enlarged images of sieve plates are shown in the lower row. Scale bar = 10 µm (upper), 2.5 µm (lower). F,G) The number (F) and diameter (G) of sieve pores in the petioles of 11‐day‐old Col‐0, *PME5OE* and *PMEI1OE* seedlings (n ≥ 6 sieve plates from at least 5 plants per genotype. The data are the sum of two independent experiments). I,J) The number (I) and diameter (J) of sieve pores in the stems of 35‐day‐old Col‐0, *PME5OE* and *PMEI1OE* plants (n ≥ 10 sieve plates from at least 5 plants per genotype. The data are the sum of two independent experiments). Error bars represent SD. Lowercase letters indicate significantly different groups as determined by one‐way ANOVA with post‐hoc Tukey's test (*p* < 0.05).

Considering that phloem is composed of sieve elements (SEs), sieve plates, companion cells (CCs), and plasmodesmata (PD), we explored whether alteration of cell wall properties affects the formation of their structures. SE‐ENOD, a monoclonal antibody recognizing SE‐localized Arabidopsis early nodulin (ENOD)‐like protein,^[^ ^51^ ^]^ was used to specifically immunolabel SEs and allowed us to count the number of SE cells and measure their diameters. We found that there were more SEs in *PME5OE* than in Col‐0, but fewer SEs in *PMEI1OE* petioles (**Figure** **4**A,B). Immunolabelling of inflorescence stems revealed the same phenomenon as observed in petioles (Figure 4C,D). SE diameter was 75.86 ± 16.40 µm in Col‐0 stems, whereas it was 98.07 ± 13.26 µm in *PME5OE* and 52.91 ± 8.87 µm in *PMEI1OE*. These results imply that pectin de‐methyl‐esterification is more conducive to SE differentiation and expansion, whereas high methyl‐esterified pectin suppresses SE development.

Perforated walls, designated sieve plates, develop at cell junctions during SE differentiation, establishing symplastic continuity for SEs in the sieve tube.^[^ ^52^ ^]^ To dissect sieve plate anatomy, we used confocal laser scanning microscopy to observe vibratome sections of petioles and inflorescence stems after fixation and Pseudo‐Schiff propidium iodide staining (mPS‐PI).^[^ ^53^ ^]^ In petioles of 11‐day‐old plants, the average diameter of sieve pores in *PME5OE* (0.57 ± 0.07 µm) was significantly larger than in Col‐0 (0.42 ± 0.03 µm), whereas it was smaller in *PMEI1OE* (0.25 ± 0.06 µm) (Figure 4E,G). These differences in sieve pore size were also observed in the stems of adult plants (Figure 4H,J). The numbers of sieve pores in both petioles and stems did not differ among the three genotypes (Figure 4F,I). These results indicate that pectin de‐methyl‐esterification facilitates sieve pore enlargement, while inhibition of de‐methyl‐esterification likely restricts sieve pore opening.

### 2.5 HG De‐Methyl‐Esterification Facilitates the Formation and Opening of PD, Boosting Cell‐to‐Cell Communication

**Figure 5:**
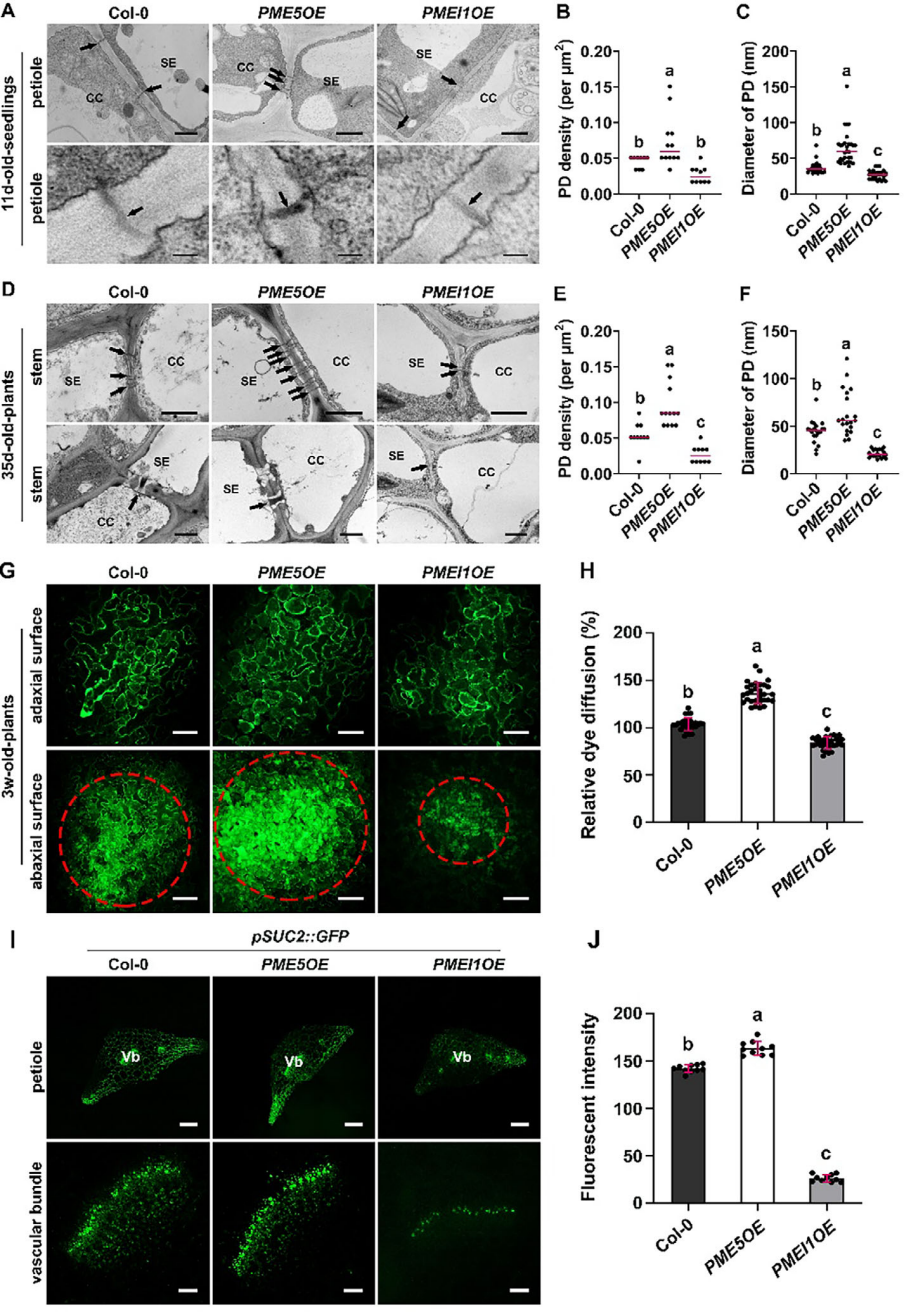
Dynamics of HG methyl‐esterification regulates plasmodesmatal architecture and intercellular trafficking in phloem. A) PD ultrastructure in the petioles of 11‐day‐old Col‐0, *PME5OE* and *PMEI1OE* seedlings. Black arrows show PD. CC, companion cell; SE, sieve element. Scale bar = 1 µm (upper), 100 nm (lower). B) PD density in Col‐0, *PME5OE* and *PMEI1OE* seedlings observed in (A). The median values are indicated by magenta lines (n = 10 regions from at least 3 seedlings for each genotype. The data are the sum of two independent experiments). C) Diameter of PD in Col‐0, *PME5OE* and *PMEI1OE* petioles described in (A) (n ≥ 25 PD per genotype. The data are the sum of two independent experiments). D) PD ultrastructure in inflorescence stems of 35‐day‐old Col‐0, *PME5OE* and *PMEI1OE* plants. Scale bar = 1 µm. E) PD density in Col‐0, *PME5OE* and *PMEI1OE* plants described in (D) (n = 10 regions from at least 3 plants. The data are the sum of three independent experiments). F) Diameter of PD in Col‐0, *PME5OE* and *PMEI1OE* stems described in (D) (n ≥ 25 PD per genotype. The data are the sum of three independent experiments). G) Confocal images showing CFDA loaded onto adaxial surfaces (upper) of 3‐week‐old Col‐0, *PME5OE* and *PMEI1OE* leaves. CF movement was observed in abaxial surfaces (lower). Red circles show the extent of dye diffusion. Scale bar = 100 µm. H) The dye diffusion was quantified by measuring the diameter of diffusion area of fluorescent dye (n ≥ 30 plants per genotype. The data are the sum of three independent experiments). I) Images of petiole sections from 11‐day‐old Col‐0, *PME5OE* and *PMEI1OE* seedlings expressing *pSUC2::GFP*, respectively. The enlarged images of vascular bundle are shown in lower row. Vb, vascular bundle. Scale bar = 200 µm (upper), 40 µm (lower). J) Fluorescent intensity of GFP in vascular bundles of petioles from transgenic plants expressing *pSUC2::GFP* (n = 10 from at least 5 petioles for each genotype. Data are representative of three biological replicates). Error bars indicate SD. Lowercase letters indicate significantly different groups as determined by one‐way ANOVA with post‐hoc Tukey's test (*P* < 0.05).

Companion cells and sieve elements, as two key components of phloem, are connected by PD, which cross cell walls and form symplastic connections between neighboring cells.^[^ ^54^ ^]^ We took a close look at PD in the wall using transmission electron microscopy (TEM) and found that *PME5OE* seedlings had more PD with wider apertures, whereas PD were sparser and narrower in the walls of *PMEI1OE* petioles (**Figure** **5**A–C). In stems, the density and diameter of PD were also higher and lower in *PME5OE* and *PMEI1OE* plants, respectively, relative to Col‐0 controls (Figure 5D–F).

To investigate whether these changes in PD architecture affect cell‐cell permeability, we performed a dye‐loading assay using carboxy‐fluorescein diacetate (CFDA) as a symplastic tracer.^[^ ^55^ ^]^ In this assay, a drop of nonfluorescent CFDA was loaded onto the adaxial epidermal surface of an intact leaf, and the symplastic diffusion of fluorescent carboxy‐fluorescein (CF) released by cellular acetylesterases was observed in the abaxial epidermis. The diameter of the fluorescent area in the abaxial epidermis indicates the extent of CF movement and PD permeability. Compared with Col‐0 controls, CF diffusion efficiency was enhanced by 40% in *PME5OE* but reduced by 15% in *PMEI1OE* leaves (Figure 5G,H). It thus appears that HG de‐methyl‐esterification is beneficial for the formation of PD in phloem, which in turn facilitates cell‐to‐cell communication and the transport of molecules and nutrients in the phloem sap.

Due to its free diffusion in phloem and neighboring tissue, *GFP* driven by the *SUC2* promoter is used as a reporter of phloem establishment and symplastic conductivity.^[^ ^51^, ^56^ ^]^ We transformed *pSUC2::GFP* into the three genotypes and examined the status of symplastic movement of molecules between cells by quantifying GFP fluorescent intensity. Compared with Col‐0 controls, GFP fluorescence was significantly higher in vascular tissue in *PME5OE* petioles, whereas it was relatively lower in *PMEI1OE* petioles (Figure 5I,J). These results indicate that alterations of HG methyl‐esterification status affect symplastic trafficking, with this being enhanced in *PME5OE* plants but reduced in *PMEI1OE* plants.

### 2.6 Methyl‐Esterification Levels of Pectic HG Are Directly Proportional to Wall Modulus in Phloem Cell Walls

**Figure 6:**
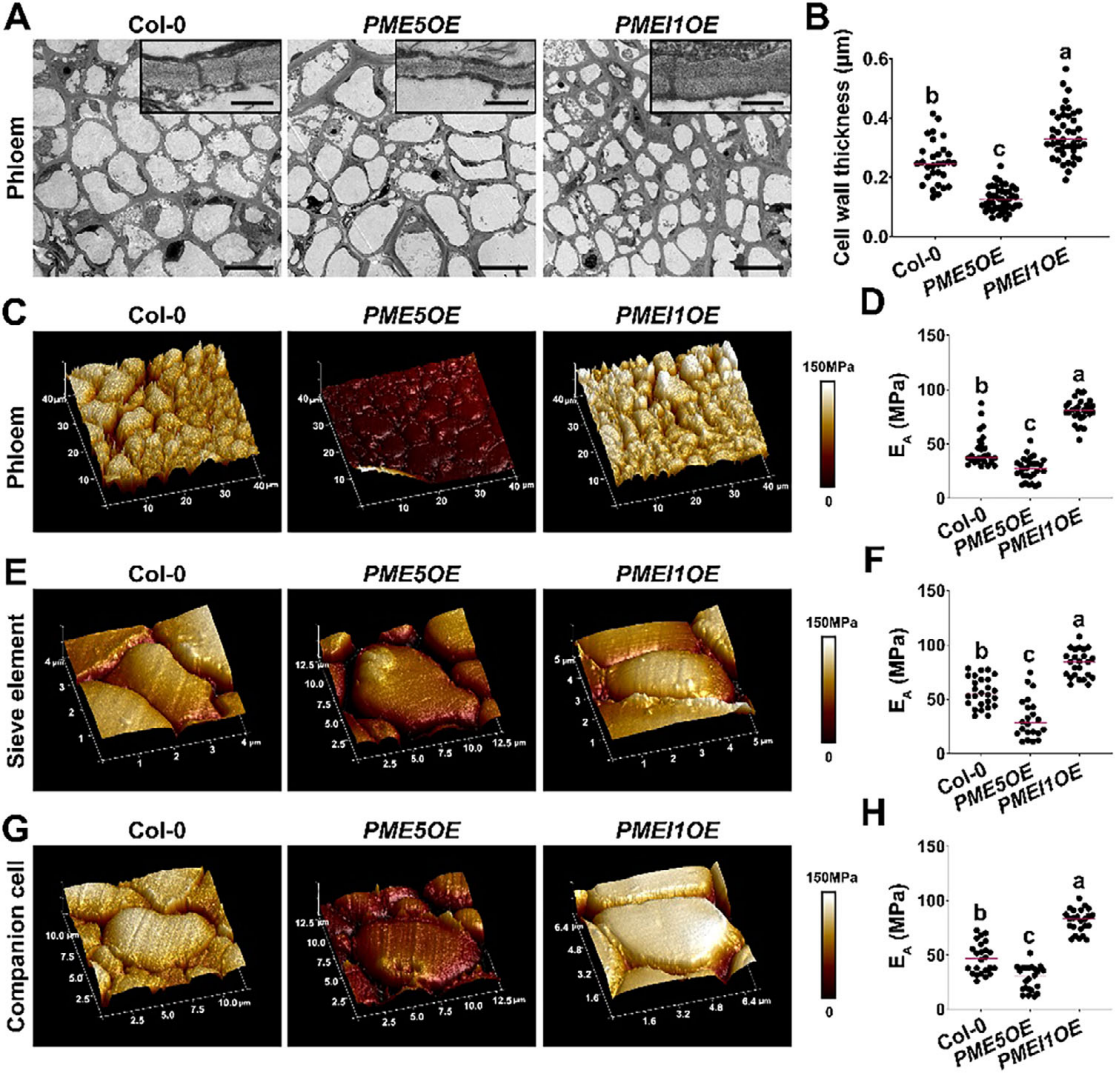
Pectin modification affects cell wall mechanical property. A) Transmission electron micrographs of stem cross‐sections from 35‐day‐old Col‐0, *PME5OE* and *PMEI1OE* plants. Scale bar = 10 µm. Details of cell wall structure are shown in insets at top right, scale bar = 500 nm. B) The thickness of cell walls observed in (A) (more than 20 cells from three plants for each genotype were used for measurement. Data are representative of three biological replicates). C,E,G) Representative maps of the apparent Young's modulus (E_A_) analyzed by AFM using the Hertzian model in fixed stem sections of 35‐day‐old Col‐0, *PME5OE* and *PMEI1OE* plants. Images are shown for the analysis in phloem (C), sieve element (E) and companion cell (G). Three sections from three different plants were analyzed. D,F,H) Quantification of apparent E_A_ obtained from AFM analysis in the cell walls of phloem (D), sieve element (F) and companion cell (H). At least 22 individual E_A_ values were taken from the elasticity map to calculate the mean and standard deviation. Lowercase letters indicate significantly different groups as determined by one‐way ANOVA with post‐hoc Tukey's test (*p* < 0.05).

Given the role of pectin chemistry in modulating cell wall mechanics,^[^ ^18^ ^]^ we investigated the cell wall properties of phloem cells in inflorescence stem cross‐sections. TEM images showed that cell walls in *PME5OE* plants were thinner than in Col‐0 plants, whereas walls were thicker in *PMEI1OE* plants than in Col‐0 plants (**Figure** **6**A,B). We next used atomic force microscopy (AFM) indentation to investigate the mechanical properties of phloem cells in transverse sections of Col‐0, *PME5OE* and *PMEI1OE* stems, in which a Hertzian model was used to fit the AFM indentation data and define Young's modulus (E_A_, the apparent coefficient of elasticity) for the tissue.^[^ ^57^ ^]^ The higher the E_A_, the stiffer (or less easily deformable) the tissue is. The walls of phloem cells in *PME5OE* plants had a lower Young's modulus than those in Col‐0, whereas *PMEI1OE* plants had a higher Young's modulus than Col‐0 (Figure 6C,D). We further analyzed the mechanical properties of sieve elements and companion cells in the phloem and found that Young's modulus was lower in *PME5OE* plants, but higher in *PMEI1OE* plants (Figure 6E–H). These results indicate that increasing HG methyl‐esterification correlates with increasing wall stiffness in phloem. It is thus likely that SEs with low HG esterification are more elastic and expand more easily.

### 2.7 Expression of Marker Genes for Phloem Differentiation was Changed in PME5OE and PMEI1OE Plants

Cell wall mechanics play a key role in maintaining cell shape and organ morphogenesis.^[^ ^58^ ^]^ The above observations prompted us to investigate whether altered wall mechanical properties lead to changes in the genetic regulation of phloem morphogenesis. Phloem development is modulated complexly at the transcriptional level, and multiple transcription factors function in the development of protophloem sieve elements.^[^ ^59^ ^]^ DNA‐BINDING ONE ZINC FINGER (DOF) transcription factors PHLOEM EARLY DOF 1 (PEAR1) and PEAR2 act to control cell divisions in developing protophloem sieve elements.^[^ ^60^ ^]^ Cell wall damage generated from the modification of cellulose and pectins activates DOF6 and TARGET OF MONOPTEROS 6 (TMO6) to promote phloem reconnection and tissue regeneration.^[^ ^61^ ^]^ ALTERED PHLOEM DEVELOPMENT (APL), a key transcriptional regulator of protophloem sieve element differentiation, is required for phloem identity.^[^ ^62^ ^]^ NAC DOMAIN‐CONTAINING PROTEIN 20 (NAC020), SUPPRESSOR OF MAX 2‐1‐LIKE 3 (SMXL3), SMXL4 and SMXL5 act as key promoters of phloem formation.^[^ ^63^, ^64^ ^]^ Thus we measured the transcript levels of these genes in Col‐0, *PME5OE* and *PMEI1OE* plants. The RT‐qPCR results showed that *PEAR1*, *NAC020*, and *SMXL5* were upregulated in *PME5OE* seedlings, whereas *PEAR1*, *PEAR2*, *APL*, *NAC020*, *SMXL3* and *SMXL5* were down‐regulated in *PMEI1OE* seedlings, although the expression of *DOF6*, *TMO6* and *SMXL4* remained unchanged across the three lines (Figure S3, Supporting Information). These data support the hypothesis that chemical modifications of pectin affect the expression levels of transcription factors required for phloem formation.

### 2.8 Functions of Pectin Modification in Phloem Development and Flowering are Corroborated in CRISPR/Cas9 Gene‐Edited Mutants

To further validate the roles of HG methyl‐esterification in Arabidopsis flowering and phloem development, we generated single mutants of *PME5* or *PMEI1* using a CRISPR/Cas9 gene‐editing strategy (Figure S4A–C, Supporting Information). Both the *pme5* and *pmei1* mutants contained single nucleotide insertions in the coding region that should result in premature termination. There was no difference in flowering time comparing the single mutants with wild type plants (Figure S4D–F, Supporting Information). We then generated CRISPR/Cas9‐mediated double mutations of *PME5* and *QRT1*, and *PMEI1* and *PMEI2*, which are closely related and located in the same clade in phylogenetic trees (Figure S5, Supporting Information). Immunolabelling experiments showed that *pme5 qrt1* mutant lines had a lower JIM5 fluorescent signal and a higher JIM7 fluorescent signal, and *pmei1 pmei2* mutant lines displayed the opposite phenotypes (Figure S6, Supporting Information). We observed that *pme5 qrt1* mutant plants flowered late, and *pmei1 pmei2* mutants flowered early compared with Col‐0 controls under both LD and SD conditions (Figure S7, Supporting Information). These results confirm that alterations of HG methyl‐esterification levels affect bolting time.

Next, we examined whether phloem architecture was altered in the double mutant plants. Compared with Col‐0 controls, cell numbers per vascular bundle in stems were significantly reduced in *pme5 qrt1* mutants; in contrast, cell numbers increased in *pmei1 pmei2* mutants. In addition, phloem cells in *pme5 qrt1* were smaller, but larger in *pmei1 pmei2* compared to wild type (Figure S8, Supporting Information). Together, these data further demonstrate that the degree of HG methyl‐esterification influences phloem development.

### 2.9 Pectin Degradation Promotes Phloem Formation and Plant Flowering

**Figure 7:**
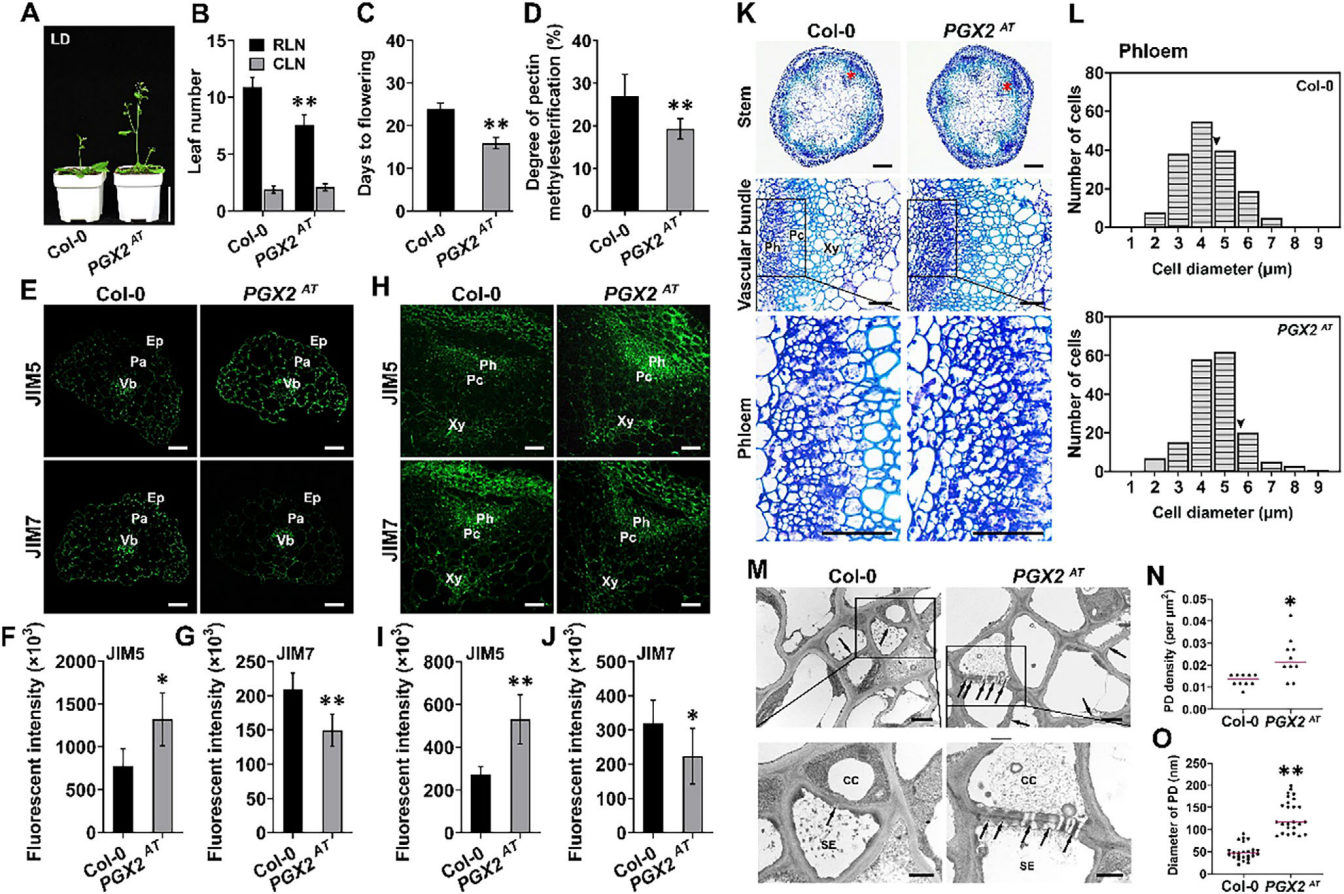
*PGX2* activation tag plants display early‐flowering, well‐developed phloem and plasmodesmata. A) 30‐day‐old Col‐0, *PGX2^AT^* plants grown in LD conditions. Scale bar = 5 cm. B) Numbers of rosette leaves (RLN) and cauline leaves (CLN) of Col‐0 and *PGX2^AT^* plants (n ≥ 30 plants per genotype. Data are representative of three biological replicates). C) Days to flowering of Col‐0 and *PGX2^AT^* plants (n ≥ 30 plants per genotype. Data are representative of three biological replicates). D) Degree of pectin methyl‐esterification in 6‐day‐old Col‐0 and *PGX2^AT^* seedlings (n = 5. Data are representative of three biological replicates). E) Immunolabelling with JIM5 and JIM7 antibodies in petiole transverse sections of 11‐day‐old Col‐0 and *PGX2^AT^* seedlings. Ep, epidermis; Pa, parenchyma; Vb, vascular bundle. Scale bar = 40 µm. F,G) Fluorescent intensity of the immunolabelling images described in (E) (n ≥ 6 images from at least 3 seedlings per genotype. Data are representative of three biological replicates). H) Immunolabelling with JIM5 and JIM7 antibodies in transverse sections of inflorescence stems from 35‐day‐old Col‐0 and *PGX2^AT^* plants. Ph, phloem; Pc, procambium; Xy, xylem. Scale bar = 40 µm. I,J) Fluorescent intensity of the immunolabelling images described in (H) (n ≥ 6 images from at least 3 seedlings per genotype. Data are representative of three biological replicates). K) Transverse sections of basal inflorescence stems in 35‐day‐old Col‐0 and *PGX2^AT^* plants. Close‐up images of the vascular bundles marked by red asterisks and phloem regions indicated by black boxes are shown in middle and lower row, respectively. Scale bar = 200 µm (upper), 50 µm (middle), 50 µm (lower). L) Distribution of the diameters of phloem cells in inflorescence stems from 35‐day‐old Col‐0 and *PGX2^AT^* plants. Arrowheads show the average diameter of phloem cells (Col‐0, n = 165; *PGX2^AT^*, n = 172). Data are representative of two biological replicates. M) Plasmodesma ultrastructure of basal inflorescence stems in 35‐day‐old Col‐0, *PGX2^AT^* plants were observed by TEM. Arrows mark PD positions. CC, companion cell; SE, sieve element. Scale bar = 2 µm (upper panel), 1 µm (lower panel). N) Plasmodesma density in Col‐0 and *PGX2^AT^* stems observed in (M). The median values are indicated by magenta lines (n = 10 regions from at least 3 seedlings. Data are representative of two biological replicates). O) Diameter of plasmodesmata in Col‐0 and *PGX2^AT^* stems observed in (M) (n ≥ 25 plasmodesmata were analyzed per genotype. Data are representative of two biological replicates). The median values are indicated by magenta lines. Data are shown as mean ± SD. *p < 0.05, **p < 0.001, Student's *t*‐test.

In the wall, de‐methyl‐esterified HG is susceptible to degradation by enzymes such as polygalacturonases. POLYGALACTURONASE INVOLVED IN EXPANSION2 (PGX2) has been reported to function in multiple developmental processes.^[^ ^34^ ^]^ Notably, *PGX2* activation tag (*PGX2^AT^*) plants flower early (**Figure** **7**A–C). Through biochemical analysis, we found that the cell walls of *PGX2^AT^* plants had decreased methyl‐esterification levels, which was consistent with immunolabelling results using JIM5 and JIM7 antibodies in both petioles and stems (Figure 7D–J). These findings reveal that the *PGX2^AT^* line phenocopies the *PME5OE* line in terms of flowering time, supporting a link between HG de‐methyl‐esterification, HG degradation, and plant flowering.

The above results piqued our interest to further investigate phloem structure in *PGX2^AT^* plants. The phloem tissue in the stems of *PGX2^AT^* lines was larger than that in Col‐0, and the average diameter of phloem cells in *PGX2^AT^* lines was also larger (Figure 7K,L). TEM images showed that PD numbers were significantly higher, and PD channels in the walls of *PGX2^AT^* were wider, than those in Col‐0 (Figure 7M‐O). These phloem phenotypes in *PGX2^AT^* plants resembled those in *PME5OE* plants. Therefore, we propose that pectin de‐methyl‐esterification and consequent degradation in the wall facilitate phloem development, which enhances the transport efficiency of molecules in the phloem including the florigen FT, promoting plant flowering.

### 2.10 Pectin Modification Influences Auxin to Regulate Phloem Development

**Figure 8:**
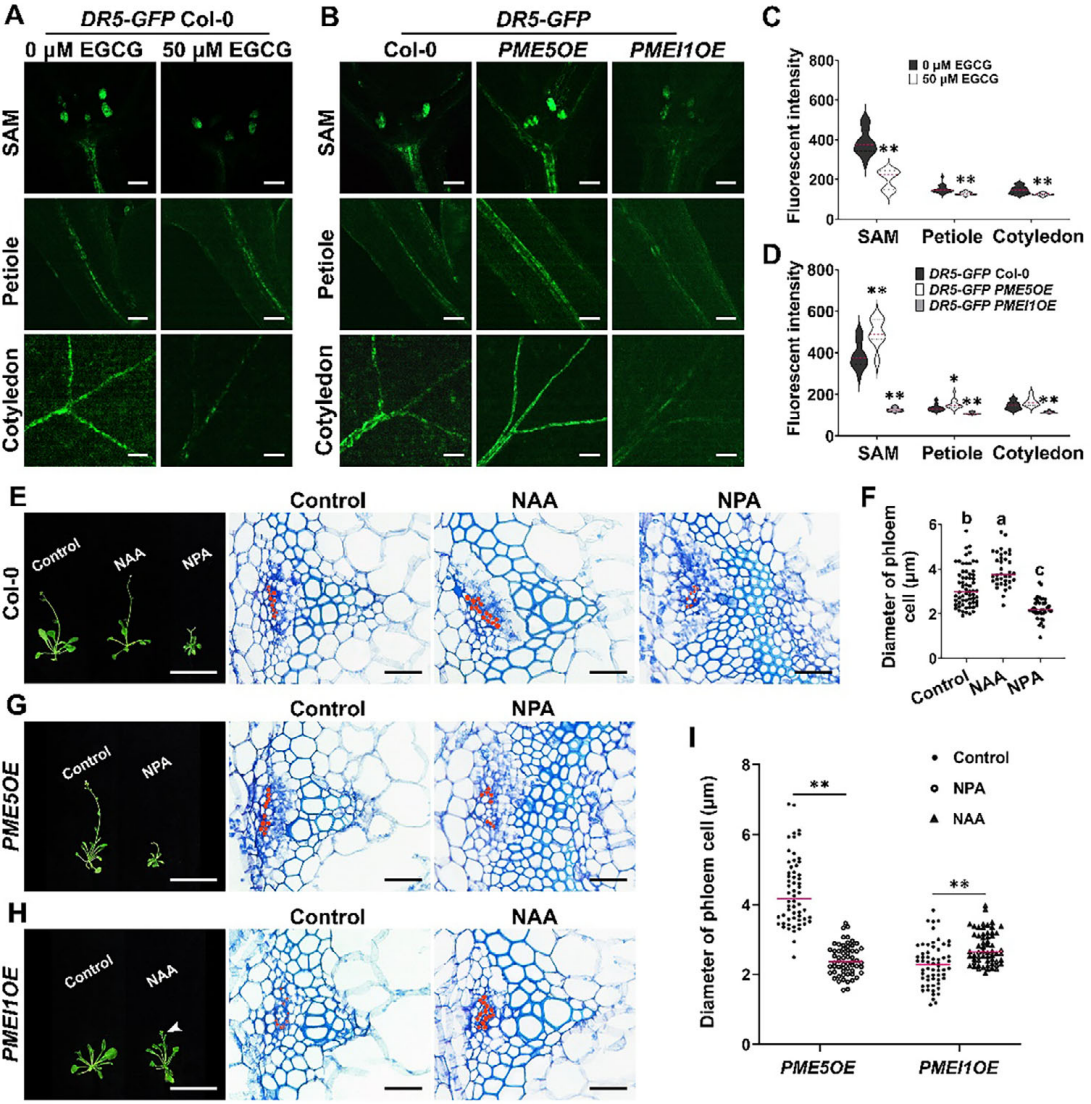
Auxin is involved in the regulation of HG methyl‐esterification status on phloem development. A) Confocal images of 4‐day‐old *DR5‐GFP* Col‐0 seedlings grown on 1/2 MS medium with or without EGCG for 2 days. Scale bar = 100 µm. B) Confocal images of 6‐day‐old Col‐0, *PME5OE* and *PMEI1OE* plants transformed with *DR5‐GFP*. Scale bar = 100 µm. C) Fluorescent intensity of DR5‐GFP in the SAM, petiole and cotyledon observed in (A) (n ≥ 20 seedlings. Data are the sum of three independent experiments). D) Fluorescent intensity of DR5‐GFP in the SAM, petiole and cotyledon observed in (B) (n ≥ 20 seedlings. Data are the sum of three independent experiments). E) Col‐0 plants grown for 5 weeks on 1/2 MS medium with or without 50 nM NAA or 20 µM NPA treatment (Scale bar = 5 mm) and transverse sections obtained from basal inflorescence stems (Scale bar = 30 µm). Red dots indicate phloem cells. F) Diameter of phloem cells of inflorescence stems in 5‐week‐old Col‐0 plants under different treatments (n ≥ 36 from at least 3 inflorescence stems for each genotype. Data are representative of three biological replicates). G) *PME5OE* plants grown for 5 weeks on 1/2 MS medium with or without 20 µM NPA treatment (Scale bar = 5 mm) and transverse sections obtained from basal inflorescence stems (Scale bar = 30 µm). H) *PMEI1OE* plants grown for 5 weeks on 1/2 MS medium with or without 50 nM NAA treatment (Scale bar = 5 mm) and transverse sections obtained from basal inflorescence stems (Scale bar = 30 µm). Arrowhead marks the bolting of *PMEI1OE* plants grown on medium supplemented with NAA. I) Diameter of phloem cells of inflorescence stems in 5‐week‐old *PME5OE* and *PMEI1OE* under different treatment conditions. The median values are indicated by magenta lines (n = 60 from at least 3 inflorescence stems for each genotype. Data are representative of three biological replicates). *p < 0.05, **p < 0.001, Student's *t*‐test. Lowercase letters indicate significantly different groups as determined by one‐way ANOVA with post‐hoc Tukey's test (*p* < 0.05).

Based on the above data, new questions arise as to how HG modifications achieve their effects on phloem development. Given that auxin is a major positional signal for vascular tissue formation,^[^ ^65^ ^]^ we explored whether HG methyl‐esterification status modulates auxin responses during phloem development. We applied epigallocatechin gallate (EGCG), an inhibitor of PME activity,^[^ ^66^ ^]^ to wild type seedlings transformed with an auxin‐responsive reporter, DR5‐GFP. EGCG treatment significantly inhibited the fluorescent signal generated from DR5‐GFP in the SAM, petiole and cotyledon (**Figure** **8**A,C). The fluorescent signal of DR5‐GFP was also significantly reduced in *PMEI1OE* seedlings, but increased in *PME5OE* seedlings, compared to Col‐0 controls (Figure 8B,D). These pharmacological and genetic data support the impingement of HG methyl‐esterification status on auxin response pathways.

We next performed chemical treatments with NAA (1‐naphthaleneacetic acid, a synthetic auxin analog) and NPA (1‐N‐naphthylphthalamic acid, an auxin transport inhibitor) in wild‐type plants. Plants grown on medium supplemented with NAA had larger phloem cells, while phloem cells were smaller when the plants were grown on medium with NPA (Figure 8E,F). To determine whether auxin impacts the expression of phloem development‐related transcription factors, we measured the expression level of marker genes in 6‐day‐old Col‐0 seedlings treated with NAA or NPA. The RT‐qPCR results showed that all tested genes had significantly increased expression levels upon NAA application (Figure S9, Supporting Information). These data suggest that enhanced auxin accumulation in *PME5OE* plants might promote phloem formation via upregulation of phloem differentiation‐related genes.

To further validate whether pectin modifications exert their effects on phloem formation via auxin signaling, we applied NPA to *PME5OE* plants and NAA to *PMEI1OE* plants and tested the effects of these agents on phloem structure. 5‐week‐old *PME5OE* plants grown on normal media produced inflorescences with flowers. However, applying NPA inhibited the growth of inflorescence stems in *PME5OE* plants, with few flowers and leaves (Figure 8G). Compared with untreated controls, the average diameter of phloem cells in NPA‐treated *PME5OE* plants was significantly smaller (Figure 8I). When NAA was applied to *PMEI1OE* plants, plants bolted earlier than untreated controls (Figure 8H). The restricted phloem cell expansion in *PMEI1OE* plants was partially rescued by NAA treatment (Figure 8I). These results suggest that pectin modifications influence auxin signaling to fine‐tune phloem formation.

## 3 Discussion

**Figure 9:**
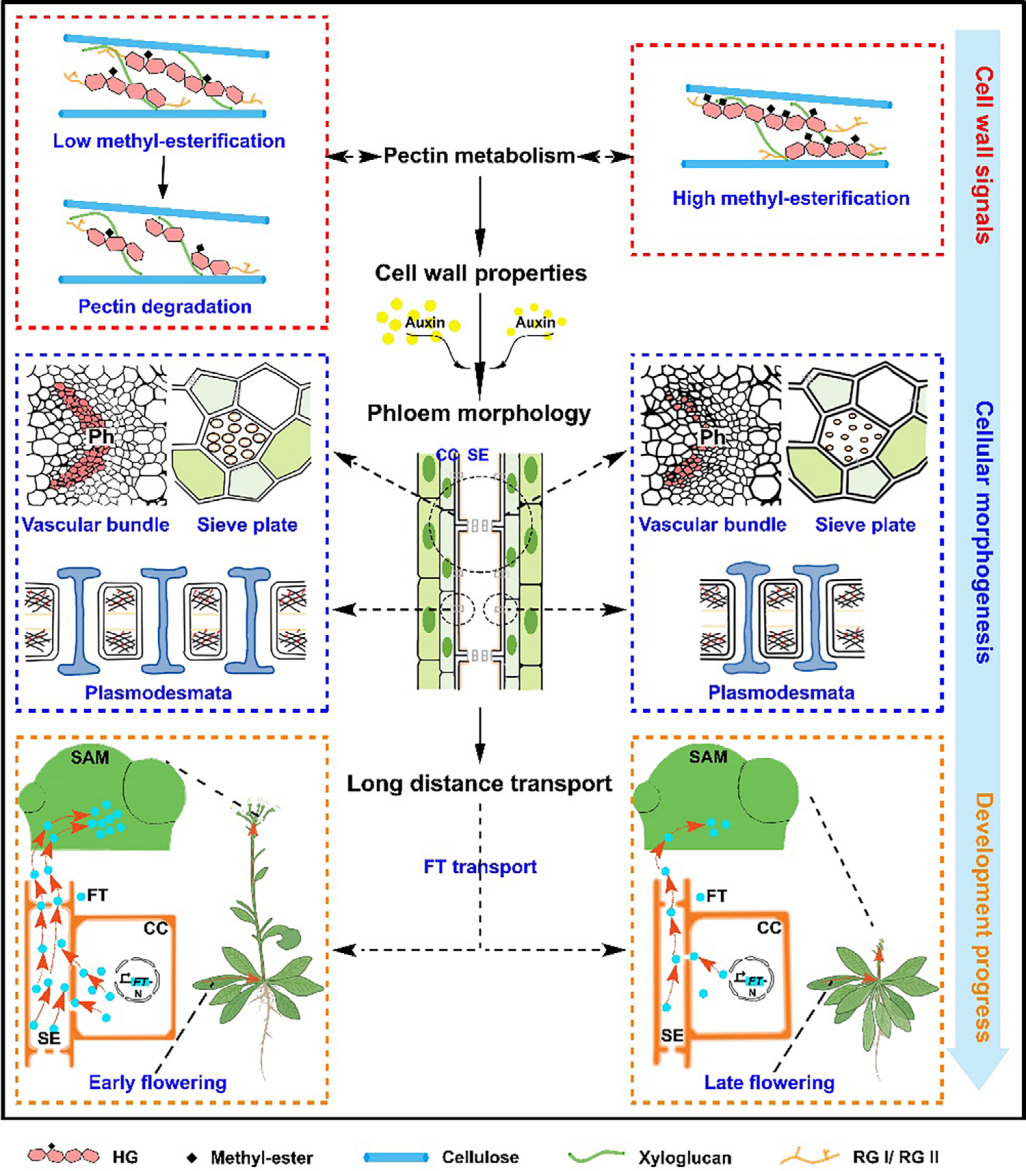
Working model showing the role of cell wall pectin metabolisms in modulating phloem morphogenesis and FT long‐distance trafficking. When PME5 and PGX2 function in cell wall, pectin de‐methyl‐esterification and consequent degradation occur, which leads to the increase of wall elasticity and the trigger of auxin responses. The decreased mechanical property and increased auxin maxima are beneficial to cell differentiation and expansion of phloem system, including sieve elements, sieve plates and plasmodesmata. These changes in phloem architecture accelerate FT transport, resulting in early flowering of plants. When pectin de‐methyl‐esterification is inhibited by PMEI1, the cell wall elasticity is reduced and auxin accumulation is suppressed, which contributes to the inhibition of phloem morphogenesis and FT movement, consequently causes plant late flowering.

Pectic HG functions in a multitude of plant developmental processes.^[^ ^7^, ^19^, ^67^, ^68^, ^69^, ^70^ ^]^ PMEs catalyze the de‐methyl‐esterification of pectic HG, making it susceptible to crosslinking or degradation, and thus play an important role in cell wall remodeling.^[^ ^71^ ^]^ De‐methyl‐esterified HG is present in rapidly expanding cells,^[^ ^72^ ^]^ and repulsive charges between pectin nanodomains have been hypothesized to result in localized wall expansion.^[^ ^21^ ^]^ We asked how pectin modifications function in the establishment and maintenance of the phloem system. In the present study, the dynamic physicochemical properties of pectic HG, and their roles in terms of wall mechanics, auxin response, phloem formation, and flowering behavior were explored, and internal functional relationships were established (**Figure** **9**). Gene expression alterations of *PME5*, *PMEI1* and *PGX2* endow cell walls with different methyl‐esterification status and mechanical properties. Auxin signaling is likely to integrate cell wall‐derived cues in modulating the formation of the phloem system. The structure of phloem determines its function: the more open the structure of the phloem, the higher the efficiency of intercellular trafficking. FT, a cargo of phloem flow, is rapidly transported from the leaves to the shoot apical meristem to induce plant flowering when the phloem conduits are unobstructed. This work revealed that pectin modifications not only have a structural role in the cell wall, but also synergistically regulate phloem morphology, thereby determining its physiological function.

The velocity and volume of cargo flow greatly depend on the geometry of the SEs and sieve plates in the phloem stream.^[^ ^31^ ^]^ Changes in the number of SE are proposed to affect phloem translocation capability (Figure 4A–D), and SE length might also contribute to the flow dynamics of the tube system. The size of sieve plate pores in *PME5OE* and *PMEI1OE* plants also differed from wild type (Figure 4E–J), implying that the sieve plates are dynamically modulated by the methyl‐esterification status of pectic HG in the wall. PD are thought of as dynamic structures, allowing for material transport and signal transduction between neighboring cells. PD channels are surrounded by pectin‐rich cell walls, and PMEs and PMEIs have both been identified in PD proteomes,^[^ ^36^ ^]^ implying a potential role of pectin modification in PD formation.^[^ ^37^ ^]^ Our results confirmed that HG de‐methyl‐esterification and degradation promoted PD formation and permeability, which were diminished in highly‐methyl‐esterified walls (Figures 5 and 7). These comprehensive dissections of the phloem system underscore the role of dynamic pectin metabolism in shaping phloem morphology and modulating intercellular communication, which may have profound impacts on adaption to changing intrinsic and environmental cues.

Mature SE cell walls contain an inner, dense pectin‐rich layer.^[^ ^33^ ^]^ A pectate lyase, PECTATE LYASE LIKE12, has been shown to function in the maturation of phloem sieve elements.^[^ ^35^ ^]^ However, the complete functions of pectins in SE cell walls are not yet clear. The role of pectin in cell wall mechanics has been investigated.^[^ ^18^ ^]^ It was reported that the methyl‐esterification status of pectic HG controls phyllotaxis by modulating wall mechanics locally.^[^ ^19^ ^]^ AFM indentation allows for the direct measurement of wall mechanics.^[^ ^58^, ^73^ ^]^ Our study highlights the utility of studying wall mechanics in internal tissues of sectioned plant materials using AFM. Walls of phloem cells including sieve elements and companion cells in *PME5OE* had a lower apparent elastic modulus, whereas the elastic modulus was higher in *PMEI1OE* plants (Figure 6C–H), supporting a mechanical function for the methyl‐esterification status of pectic HG in phloem architecture. It is noteworthy that other pectic domains (such as RG I) and polysaccharides (such as cellulose) and complex interactions between them have been detected in the wall of SE cells.^[^ ^33^ ^]^ These polymers and their interactions are also likely to affect wall elasticity in phloem cells.^[^ ^74^ ^]^ In addition, analyses of the PD proteome identified multiple peroxidases,^[^ ^36^ ^]^ which can loosen or stiffen cell walls in different developmental contexts.^[^ ^75^, ^76^ ^]^ One question that remains unsolved is how other polysaccharides and proteins contribute to the mechanical properties of the phloem system.

The co‐occurrence of HG methyl‐esterification dynamics and auxin signaling exists in different developmental processes. Auxin can trigger changes in HG methyl‐esterification and wall mechanical properties.^[^ ^77^ ^]^ However, a feedback loop between auxin and HG methyl‐esterification has been implicated in apical hook formation during seedling emergence and organ outgrowth in the shoot apical meristem.^[^ ^42^, ^43^ ^]^ In this study, we observed that overexpression of *PME5* and *PMEI1* led to enhanced and reduced auxin accumulation in the investigated tissues comparing with wild‐type plants, respectively (Figure 8B,D). Meanwhile, EGCG treatment attenuated auxin maxima in vascular cells (Figure 8A,C). In *PMEI1OE* plants, vascular bundles phenocopied those of NPA‐treated Col‐0 plants (Figures 3 and 8E,F). Notably, NPA inhibited phloem cell development in *PME5OE* plants, and NAA promoted phloem cell development in *PMEI1OE* plants (Figure 8G–I). These data suggest that HG methyl‐esterification‐mediated alterations in wall mechanics coordinate auxin signaling to regulate phloem formation in a mechano‐chemical feedforward loop. However, other possible forms of this loop should also be considered. One possibility is that oligogalacturonides released upon pectin cleavage stimulate auxin signaling and/or activate transcription factors such as DOF to regulate vascular formation.^[^ ^78^ ^]^ Indeed, several key phloem differentiation‐related regulators exhibited significant changes in expression levels in *PME5OE* and *PMEI1OE* seedlings (Figure S3, Supporting Information). The transcriptional fluctuation of these genes implies their differing roles in the initiation of phloem development. It is also possible that changes in pH in the wall modulated by pectin modifications affect auxin behavior during phloem differentiation.^[^ ^79^ ^]^

In the phloem stream, sugars, proteins and/or RNAs would be subjected to the same pressure flow that guides FT protein from the leaf to the SAM. Several pieces of evidence in this study support a function for pectin modifications in cell walls in modulating FT trafficking. First, altered expression of *PME5*, *QRT1*, *PGX2*, *PMEI1* and *PMEI2* affected flowering time (Figures 2A–C and 7A–C and Figure S7, Supporting Information). Interestingly, *PME5OE* and *PGX2^AT^* plants consistently flowered early, implying an effect of HG de‐methyl‐esterification and consequent degradation on the control of flowering time. We demonstrated that *PGX2^AT^* plants exhibit reduced HG methyl‐esterification, with a similar finding for *PME5OE* plants. The degradation state of HG in *PME5OE* plants will be interesting to explore in the future. Second, FT protein transport was accelerated in *PME5OE* seedlings, and retarded in *PMEI1OE* seedlings under control of the β‐estradiol induction system (Figure 2D–F). The timing of FT‐GFP protein unloading in the SAM was also altered in the two lines with opposite HG methyl‐esterification status (Figure 2G). Third, the difference in long‐distance transport of FT in *PME5OE* and *PMEI1OE* plants was corroborated in grafting experiments (Figure 2H,I). Finally, the anatomic profiling of phloem in *PME5OE*, *PMEI1OE*, *PGX2^AT^*, *pme5 qrt1* and *pmei1 pmei2* plants demonstrated that the HG de‐methyl‐esterification and degradation is beneficial for the formation and cell‐to‐cell communication of phloem (Figures 3, 4, 5 and 7 and Figure S8, Supporting Information). All these results point toward an underlying mechanism by which the morphology of the phloem system mediated by dynamic cell wall modifications controls FT trafficking and flowering time. This points to a new strategy for crop breeding to optimize flowering time through cell wall metabolism as an alternative to the genetic manipulation of FT.^[^ ^80^, ^81^, ^82^ ^]^ Studying the molecular mechanisms by which HG methyl‐esterification coordinates auxin signaling to regulate phloem development would be a promising direction for future investigation.

## 4 Experimental Section

### Plant Materials and Growth Conditions

*Arabidopsis thaliana* mutants used in this study are in Columbia‐0 (Col‐0) background. *PMEI1OE* and *PGX2^AT^* plants have been described previously,^[^ ^34^, ^46^ ^]^ *PME5OE* transgenic plants were generated by expressing *PME5* gene driven by *35S* promoter. *DR5‐GFP PME5OE* and *DR5‐GFP PMEI1OE* plants were generated by crossing *DR5‐GFP* plants with *PME5OE* and *PMEI1OE*, respectively. Seeds were surface sterilized in 30% (v/v) bleach containing 0.1% sodium dodecyl sulfate, and washed four times with sterile water before resuspending in 0.15% (w/v) agar. After stratification at 4 °C for at least 2 days, seeds were sown on ½ MS plates containing 2.2 g L^−1^ Murashige and Skoog salts, 0.6 g L^−1^ 2‐N‐morpholino‐ethanesulfonic acid, 1% (w/v) sucrose and 0.8% (w/v) agar, pH 5.6. Plates were placed in a growth chamber at 22 °C with a 16‐h light/8‐h dark photoperiod. Seedlings were transferred into soil and continued to grow in a greenhouse with the same temperature under long‐day condition (16‐h light/8‐h dark) or short‐day condition (8‐h light/16‐h dark).

### Phylogenetic Analyses

*Arabidopsis thaliana* PMEs^[^ ^71^ ^]^ and PMEIs^[^ ^11^ ^]^ sequences were downloaded using BLASTP (TAIR). Sequences of PMEs and PMEIs were aligned using Clustal W in MEGAX. Neighbor‐joining phylogenetic trees were built using MEGAX with 1000 bootstraps.

### Plasmid Construction and Transgenic Plants

To generate gene overexpression plants, the full‐length coding sequence of *PME5* was amplified and cloned into pGWB661 using the Gateway cloning system (Life Technologies, catalog No. 11 791 020) to generate a *35S::PME5* construct. For the generation of *pSUC2::GFP* vector, a 2129 bp upstream sequence of the *SUC2* start codon was amplified and subsequently ligated into the binary vector pH7FGW2 to replace the *35S* promoter. The 2129 bp *SUC2* promoter fragment and *FT* genomic sequence were cloned into the binary vector pH7FGW2 to generate a construct of *pSUC2::FT‐GFP*. For the development of *pKNAT1::FT‐GUS*, a 1477 bp promoter sequence of *KNAT1* and *FT* genomic sequence were integrated into pBI121. Single mutants of *pme5* and *pmei1*, as well as double mutants of *pme5 qrt1*, *pmei1 pmei2* were generated using CRISPR‐Cas9 gene editing strategy. sgRNA sequences of *PME5‐*targeting, *QRT1‐*targeting, *PMEI1‐*targeting, *PMEI2‐*targeting were designed in the CRISPR‐P 2.0 web (http://crispr.hzau.edu.cn/CRISPR2/). DNA oligonucleotides of sgRNA were annealed to generate double‐stranded DNA before ligating to the CRISPR/Cas9 expression vector pHSE401. All primer sequences for vector construction are listed in Table S1, Supporting Information. Plant transformation was performed using the floral dipping method.^[^ ^83^ ^]^ Positive transgenic seedlings were screened on ½MS medium containing appropriate antibiotics (25 mg mL^−1^ hygromycin, 50 mg mL^−1^ kanamycin, or 25 mg mL^−1^ L‐methionine sulfoximine).

### RNA Extraction and RT‐qPCR

Total RNA was extracted by using a Plant RNA Kit (Omega, catalog No. R6827) by following the manufacture's instructions. One microgram of total RNA was subjected to treatment with gDNA Eraser (Takara) to remove genomic DNA. An aliquot of 1 µg RNA for each sample was used for reverse transcription using the PrimeScript RT reagent Kit (Takara, catalog No. RR047A). The RT‐qPCR assays were performed using the TB Green Ex Taq II (Takara, catalog No. RR420A) with a real‐time fluorescence quantitative PCR system (BioRad). The expression level of each gene was normalized to *ACT2*. All of the reactions were performed three times independently. A list of the primers used for RT‐qPCR is provided in Table S2, Supporting Information.

### Measurements of Uronic Acids and HG Methyl‐Esterification Levels

The measurement of total uronic acids was performed as previously described with minor modifications.^[^ ^84^ ^]^ Briefly, alcohol‐insoluble residue (AIR) was extracted from 6‐day‐old light‐grown seedlings following a published procedure.^[^ ^85^ ^]^ Samples of 1 mg AIR were suspended in 0.4 mL of water in a glass tube. Forty microliters of 4 M sulfamic acid‐potassium sulfamate (pH 1.6) was added and mixed thoroughly. Then 2.4 mL of concentrated H_2_SO_4_ containing 75 mM sodium tetraborate (Sigma, catalog No. 221 732) was added and mixed vigorously by vortexing for 10 s. The solution was heated in boiling water for 10 min. After cooling down to room temperature, eighty microliters of 0.15% m‐hydroxydiphenyl (Sigma, catalog No. 262 250) in 0.5% NaOH was added and vortexed. Following reaction for 5 min, the absorbance of 525 nm was measured. A standard curve of D‐GalA (Sigma) and absorbance was used to calculate uronic acid content (nmol mg^−1^).

Following the previous procedure, the released methylesters were measured.^[^ ^25^, ^86^ ^]^ About 1 mg of AIR was weighed and used for analysis. After washing in 1 mL of water and centrifugation at 15 000 rpm (Eppendorf Centrifuge 5424) for 10 min at room temperature, four hundred microliters of 0.5 M NaOH was added and incubated at room temperature for 1 h. Then 200 µL of 1 M HCl was added for neutralization. Samples were centrifuged at 5000 rpm (Eppendorf Centrifuge 5424) for 10 min at room temperature. An aliquot of 200 µL supernatant was mixed with 300 µL of 20 mM HEPES buffer (pH 7.5). The released methanol was oxidized by adding 500 µL of HEPES buffer containing 0.03 units of alcohol oxidase (Sigma, catalog No. A2404) and shaking for 15 min at room temperature. Then an aliquot of 500 µL of assay buffer (20 mM acetyl acetone, 50 mM acetic acid, and 2 M ammonium acetate) was added to each sample and developed at 60 °C for 15 min. After cooling, the absorbance at 412 nm was measured using a NanoDrop spectrophotometer. A series of diluted methanol aliquots were used for a standard curve to calculate total methylester content. Assuming that methanol was released primarily from pectic HG, the methyl‐esterification level of HG was estimated by dividing nmol of released methanol by nmol of uronic acids in the same amount of AIR.^[^ ^85^ ^]^

### Semi‐Thin Section Preparation

Petioles and stems were fixed with 2.5% glutaraldehyde in PBS buffer (pH 7.2) for 4 h at 4 °C. The samples were rinsed with distilled water for 5 min, and dehydrated with a series of ethanol (10%, 20%, 30%, 50%, 70%, 90% and 100%) for 10 min for each step. Tissues were infiltrated with the solution containing methacrylate mix (75% (v/v) butyl methacrylate, 25% (v/v) methyl methacrylate, 0.5% (w/v) benzoin ethyl ether and 10 mM DTT) and ethanol gradients (30%, 50%, 70% and 90%) step by step, which was followed by three 8 h incubations in methacrylate mix (resin embedding matrix). Samples were embedded in gelatin capsules by exposure to UV light for at least 12 h at room temperature. Sections were made with a Leica UC7 ultramicrotome with 1.5 µm thickness and transferred to adhesive microscope slides.

### Immunofluorescence Assays

Blocking and immunolabelling were conducted at room temperature. After blocking with 3% (w/v) bovine serum albumin for 1 h, 1:10 diluted primary antibody JIM5 (Agrisera, catalog No. AS184194) or JIM7 (Agrisera, catalog No. AS184195) was added onto the slide and incubated for 2 h. Samples were rinsed three times with PBS buffer and incubated with 1:500 diluted Alexa Fluor 488‐conjugated goat anti‐rat IgG secondary antibody (KPL, catalog No. 5230‐0340) in the dark for 1 h. Images were collected under a spinning‐disk confocal microscope with a 488‐nm excitation laser and 525/50‐nm emission filter with a 20× or 100× objectives (Zeiss, Observer SD). The fluorescent intensity of single‐plane immunolabelling images in the selected regions was quantified by ZEN software (Zeiss).

For immunostaining in the sieve element, tissues were prepared using a vibratome. Petioles and stems were sectioned after fixing for 1 h in 4% paraformaldehyde dissolved in PBS buffer containing 1% dimethylsulfoxide and 0.1% Triton X‐100. The primary antibody against the SE‐ENDOD protein of phloem sieve elements (ENOD 9, Kerafast, catalog No. EIW201) was diluted at a ratio of 1:100 and incubated for 1 h. The secondary antibody Alexa Fluor 488 goat anti‐mouse IgG (Invitrogen, catalog No. A‐11030) was diluted at a ratio of 1:100 and incubated for 1 h. Samples were rinsed with water and incubated in Schiff reagent (100 mM sodium metabisulphite and 0.15 M HCl) with 100 µg mL^−1^ propidium iodide (Life Technologies, catalog No. P3566) for 1 min before imaging under a Zeiss spinning‐disk confocal microscope with 488 and 561 nm lasers.

### Flowering Time

Flowering time was assessed by counting the number of rosette and cauline leaves on the main stem when the inflorescence reached ≈5 cm.

### Plant Protein Extraction and Immunoblotting

Total plant proteins were extracted according to the previous description.^[^ ^87^ ^]^ Western‐blot was performed with an anti‐GFP antibody (1:5000, Agrisera, catalog No. AS152987), an anti‐Actin antibody (1:5000, Agrisera, catalog No. AS132640), and HRP conjugated goat anti‐rabbit IgG (1:10 000, Agrisera, catalog No. AS09602). The signal was visualized using an ECL solution and photographed in a chemiluminescent gel imager (Bio‐rad ChemiDoc Touch).

### β‐Estradiol Induction of FT‐GFP Expression

β‐estradiol induction experiments were performed as previously described with minor modification.^[^ ^49^ ^]^ Briefly, β‐estradiol (20 µM, Sigma, catalog No. 50‐28‐2) was applied on the cotyledons of 8‐day‐old *pER8::FT‐GFP* transgenic seedlings with paint brushes. After growing for another 3 days, induced cotyledons with petioles were dissected and examined under a Zeiss confocal microscope with a 10× objective. For the observation of flowering time phenotype with β‐estradiol induction, 5‐day‐old seedlings were treated with 20 µM β‐estradiol every 3 days till bolting. To estimate how fast the FT‐GFP is unloaded onto the shoot apical region, β‐estradiol (20 µM, ≈500 µL per plant) was sprayed on the leaf blade of plants after they had bolted for 5 cm, and shoot apical regions were collected 0, 4, 8, 12, and 24 h after treatments to detect FT‐GFP protein by western blotting.

### Hypocotyl‐Grafting Experiments

The hypocotyl‐grafting surgery was performed by following a previously developed protocol,^[^ ^88^ ^]^ in which 5‐day‐old seedlings grown under LD conditions on ½MS medium without sucrose were used. After surgery, grafted seedlings were allowed to recover for 10 days. Successfully stem‐grafted plants were identified with the fluorescent signal under a confocal microscope with a 10× objective (Zeiss, Observer SD).

### Paraffin Section Preparation

Samples were cut and fixed in 50% FAA for 24 h at room temperature, dehydrated stepwise in an ethanol series from 30% to 100%, and then embedded in paraffin and sliced into 5 µm sections using a paraffin slicer (Leica).

### Toluidine Blue O Staining

Paraffin sections were de‐waxed with xylene and dehydrated through an ethanol series. For toluidine blue O staining, slides were exposed to 0.05% (w/v) toluidine blue in water (pH 5.0) for 20 s. Sections were then washed with water and photographed under an epifluorescence microscope (Leica, DM4B).

### mPS‐PI Staining and Imaging of Sieve Plates

mPS‐PI staining was performed as previously described.^[^ ^53^ ^]^ Briefly, whole seedlings or tissue organs were fixed in a fixative of 50% methanol and 10% acetic acid at 4 °C at least for 12 h. Samples were transferred to 80% ethanol and incubated at 80 °C for ≈1–5 min. Then samples were put back into fixative and incubated for 1 h followed by rinsing with water and incubation in 1% periodic acid for 40 min. After rinsing with water, samples were incubated in Schiff reagent with propidium iodide (100 mM sodium metabisulphite and 0.15 M HCl with freshly added propidium iodide to a final concentration of 100 µg mL^−1^) until staining was visible. Samples were transferred onto microscope slides and covered with a chloral hydrate solution (4 g chloral hydrate, 1 mL glycerol and 2 mL water). Slides were kept overnight at room temperature in a closed environment to prevent drying. After removing excess chloral hydrate, a few drops of Hoyer's solution (30 g gum arabic, 200 g chloral hydrate, 15.9 mL glycerol and 50 mL water) were applied to the samples, followed by the placement of a cover slip. The slides were then left undisturbed for at least 3 days to allow the mounting solution to solidify before imaging.

For imaging of sieve plates, petiole and stem cross‐sections (100 µm in thickness) were generated with a vibratome (Leica, VT1000S). Samples were subjected to mPS‐PI staining as described above. Images were collected under a spinning‐disk confocal microscope with 100× objective (Zeiss, Observer SD).

### Quantifications of PD Number and PD Diameter

Numbers and diameters of PD were analyzed manually using ImageJ. PD density was quantified by counting the number of PD in the areas of the same size. The diameters were measured at the neck region of PD.

### PD Permeability Assays

Drop‐ANd‐See (DANS) experiments for PD permeability were performed as previously described.^[^ ^55^ ^]^ DANS dye loading assay was performed by loading 1 µL drop of 1 mM CFDA (Sigma, catalog No. 79955‐27‐4) on the adaxial side of the intact rosette leaf and incubating for 5 min. The droplets were removed and the loading region of leaf was gently rinsed with water. The leaf was then detached and immediately mounted. The fluorescent signal on the abaxial leaf was imaged under a confocal microscope with a 488 nm laser (Zeiss, Observer SD). The efficiency of dye diffusion was quantified by measuring the diameter of the diffusion area for fluorescence distribution.

### EGCG Treatment

EGCG, as a natural inhibitor of PME activity, has been widely used in the characterization of pectin methylesterases,^[^ ^66^, ^89^, ^90^ ^]^ although EGCG application might affect localization and dynamics of several PM‐localized proteins.^[^ ^91^ ^]^ In this experiment, 4‐day‐old seedlings vertically grown on ½ MS medium plates were transferred to solid ½ MS medium supplemented with 50 µM EGCG (Sigma, catalog No. 989‐51‐5) and continued to grow for 2 days. Samples were placed in distilled water to observe under a spinning‐disk confocal microscope with a 488‐nm excitation laser (Zeiss, Observer SD).

### NAA and NPA Treatments

For NAA and NPA treatment experiments, NAA (Shenggong, catalog No. 86‐87‐3) was dissolved in 1N NaOH at 5 mM, and NPA (Aladdin, catalog No. 132‐66‐1) was dissolved in dimethylsulfoxide at 20 mM. Plants were grown on ½ MS medium supplemented with 50 nM NAA or 20 µM NPA for 5 weeks. Cross‐sections of basal inflorescence stems were photographed under an epiflorescence microscope (Leica, DM4B). For gene expression experiment, 6‐day‐old Col‐0 seedlings were grown on ½ MS medium supplemented with 0.5 µM NAA or 20 µM NPA for 12 h or 24 h.

### Transmission Electron Microscopy

For observation of PD structure by using TEM, samples of petioles and stems were cut and prefixed in a PBS buffer (pH 7.2) containing 2.5% glutaraldehyde at 4 °C overnight. The specimens were washed twice with PBS buffer and postfixed in 1% osmium tetroxide (OsO_4_) at 4 °C for 4 h. The specimens were dehydrated stepwise in an acetone series from 30% to 100% and embedded in epoxy resin (Epon812). Ultrathin sections (≈60–90 nm in thickness) were obtained by using a Leica UC7 ultramicrotome, and stained with 2% uranyl acetate and lead citrate. Images were photographed with a TEM (JEM‐1400FLASH).

### Atomic Force Microscopy

Transverse sections of fixed Arabidopsis stems were prepared as described above. The samples were imaged by AFM (ICON, Bruker) using PeakForce QNM mode in the air in ambient conditions. The standard pyramidal silicon nitride probes (ScanAsyst‐Air, Bruker) with a nominal spring constant of 2.6 N m^−1^ and a nominal tip radius of 2 nm were applied for AFM imaging at different amplitude setpoints to optimize feedback gain values. Experimental data were acquired from three phloem regions of different sections. In each region, a minimum of five companion cells and sieve element cells were examined. Apparent Young's modulus was quantified by using NanoScope software (version 3.0) according to the previously described method.^[^ ^33^, ^92^ ^]^

### Confocal Imaging

Petiole cross‐sections with a thickness of 100 µm were cut from 11‐day‐old *pSUC2::GFP* seedlings by using a vibratome (Leica VT1000S). Petiole sections were placed on the glass slides and the GFP signal was observed under a spinning‐disk confocal microscope with a 488 nm laser (Zeiss, Observer SD). 4 or 6‐day‐old seedlings of *DR5‐GFP* were cultured in ½ MS medium plates. The GFP signal was observed using a spinning‐disk confocal microscope with a 488 nm laser (Zeiss, Observer SD).

### Statistical Analyses

All statistical analyses were performed using GraphPad Prism 9. All experiments were repeated at least three times unless otherwise stated. Data are represented as averages ± SD. Analyses were performed using Student's *t*‐test (two‐tailed) or one‐way ANOVA with Tukey's test to evaluate the significant difference. P value thresholds are shown as *P* < 0.05 or *P* < 0.001.

### Accession Numbers

Sequence data from this article can be found in the Arabidopsis Genome Initiative under the following accession numbers: PME5 (AT5G47500), QRT1 (AT5G55590), PMEI1 (AT1G48020), PMEI2 (AT3G17220), FT (AT1G65480), PGX2 (AT1G78400), PEAR1 (AT2G37590), PEAR2 (AT5G02460), DOF6 (AT3G45610), TOM6 (AT1G49410), APL (AT1G79430), NAC020 (AT1G54330), SMXL3 (AT3G52490), SMXL4 (AT4G29920), SMXL5 (AT5G57130), ACT2 (AT3G18780).

## Conflict Interest

The authors declare no conflict of interest.

## Author Contributions

C.X. and J.D. designed the research; Q.Z., J.D., S.H., Z.L., and Y.Y. performed experiments; J.D., W.L., C.Z., L.C., and C.X. supervised the project, Q.Z., J.D., and C.X. analyzed the data; and Q.Z., J.D., C.T.A., and C.X. wrote the article. Q.Z. and J.D. contributed equally to this work.

## Supporting information



Supporting Information

## Data Availability

The data that support the findings of this study are available in the supplementary material of this article.
