# Supplementary material for: Pectin Metabolism Influences Phloem Architecture and Flowering Time in Arabidopsis Thaliana
Source: Adv Sci (Weinh). 2025 Jul 29;12(39):e02980. doi: 10.1002/advs.202502980 (PMC12533402; doi:10.1002/advs.202502980)
Supplement: Supplementary file 1 — Supporting Information [file ADVS-12-e02980-s001.docx]

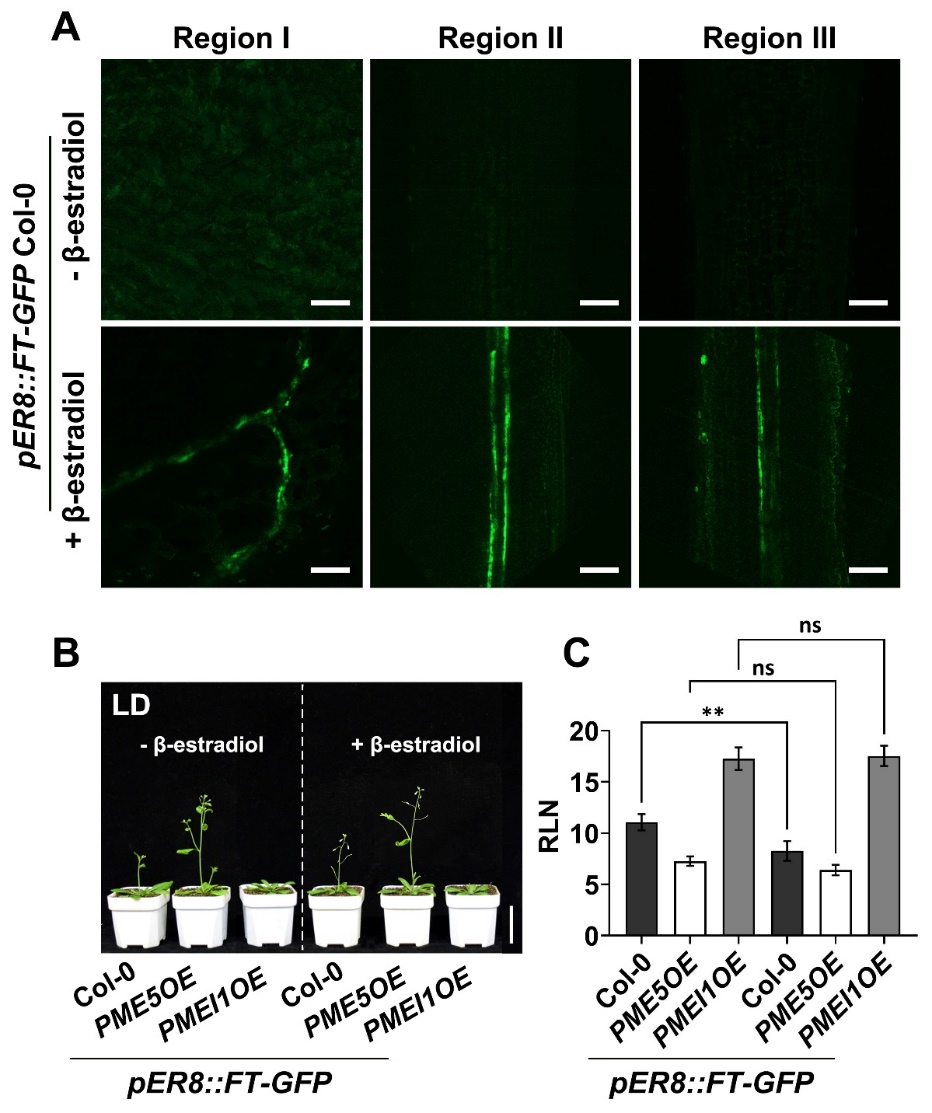


**Figure S1. β-estradiol treatment induces FT protein expression.** (**A**) FT-GFP observed in the vascular tissues of 11-day-old *pER8::FT-GFP* Col-0 transgenic plants with β-estradiol induction. Scale bars = 100 µm. (**B**) Flowering phenotypes of 4-week-old Col-0, *PME5OE* and *PMEI1OE* plants transformed with *pER8::FT-GFP* in long-day (LD) conditions with or without β-estradiol induction. Scale bar = 5 cm. (**C**) Numbers of rosette leaves (RLN) of transgenic plants containing *pER8::FT-GFP* without (left three columns) and with (right three columns) β-estradiol induction. β-estradiol (20 µM) was applied on plants every three days starting on the fifth day after seed germination until the first flower bud was visible (n ≥ 20 plants per genotype. Data are representative of three biological replicates). Error bars represent SD. ns indicates no significant difference. ***P* < 0.001, Student’s *t*-test.


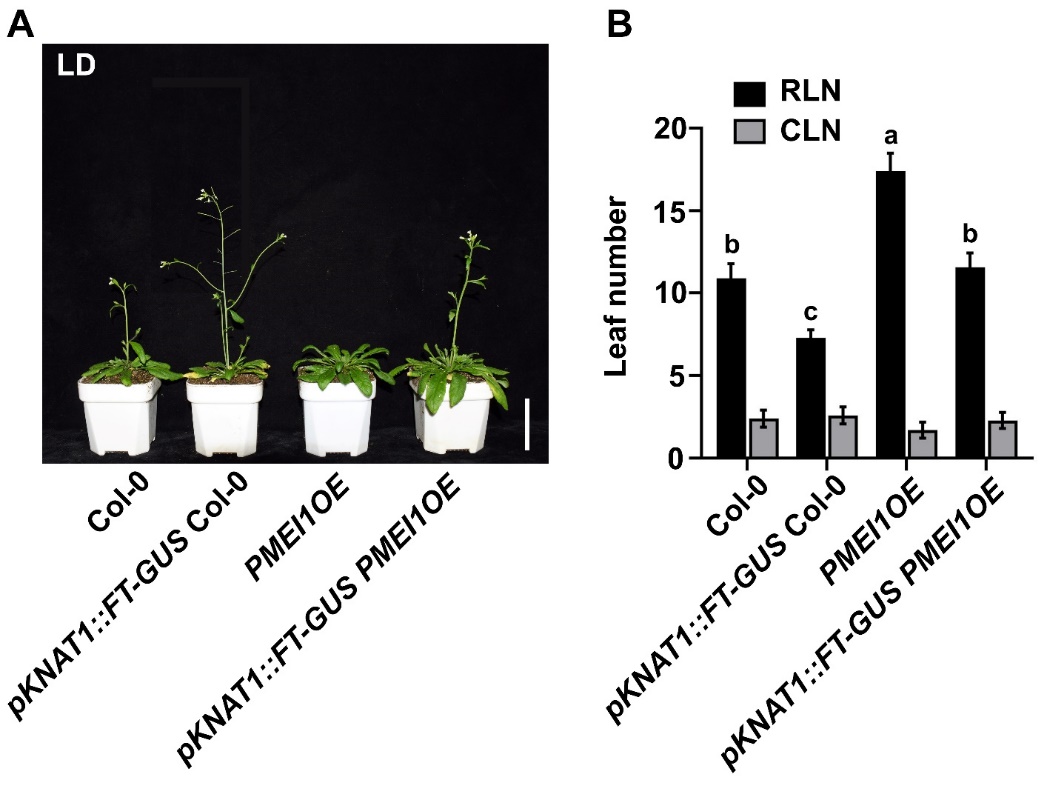


**Figure S2. Specific expression of *FT* in shoot apical meristem promotes early flowering in *PMEI1OE* plants.** (**A**) Flowering phenotypes of 30-day-old Col-0, *pKNAT1::FT-GUS* Col-0, *PMEI1OE*, *pKNAT1::FT-GUS* *PMEI1OE* plants grown in long-day (LD) conditions. Scale bar = 5 cm. (**B**) Numbers of rosette leaves (RLN) and cauline leaves (CLN) of Col-0, *pKNAT1::FT-GUS* Col-0, *PMEI1OE*, *pKNAT1::FT-GUS PMEI1OE* plants in LD conditions (n ≥ 30 plants per genotype. Data are representative of three biological replicates). Error bars represent SD. Lowercase letters on top of bar charts indicate significantly different groups as determined by one-way ANOVA with post-hoc Tukey's test (*P* < 0.05).


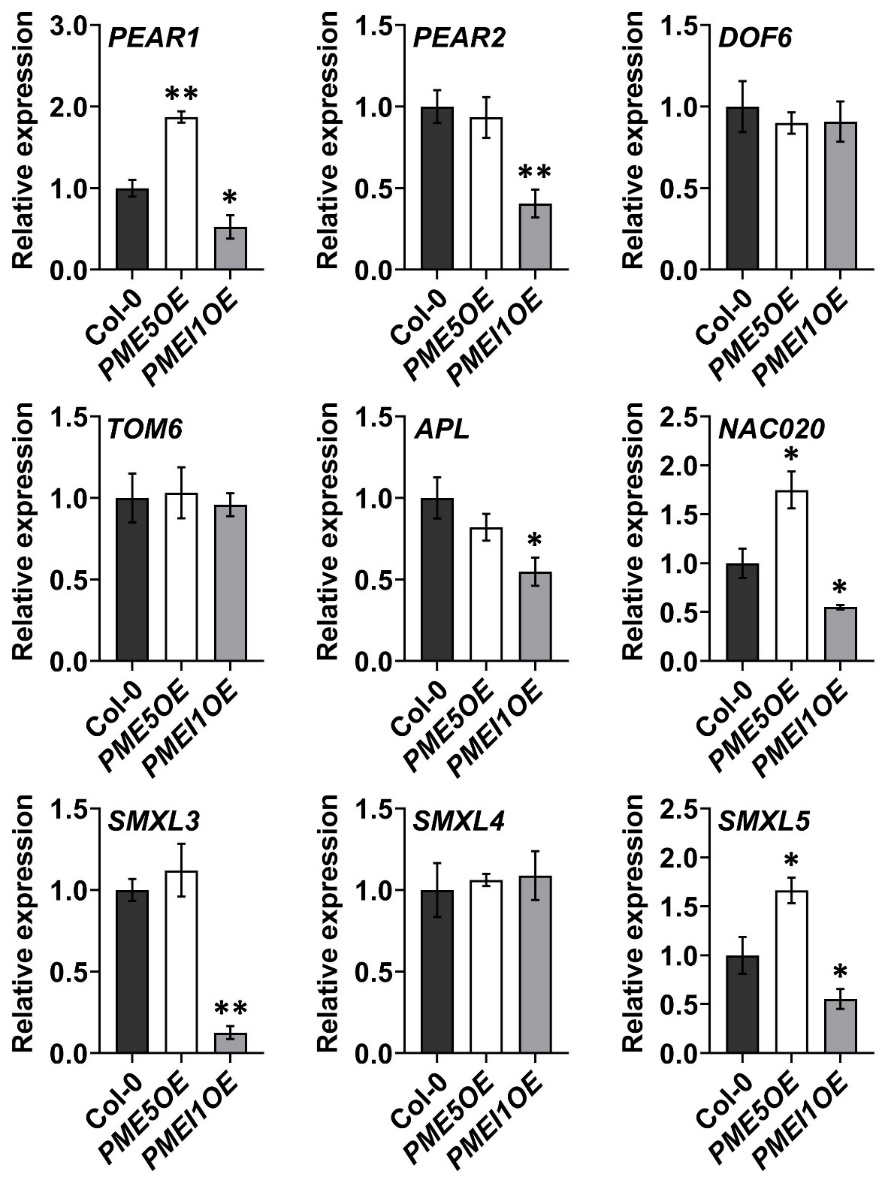


**Figure S3. Relative expression levels of phloem differentiation-related genes.** Gene expression levels were detected by RT-qPCR in 6-day-old Col-0, *PME5OE* and *PMEI1OE* seedlings (n = 3. Data are representative of three biological replicates). *ACT2* was used as an internal control. Error bars represent SD. **P* < 0.05, ***P* < 0.001, Student’s *t*-test.


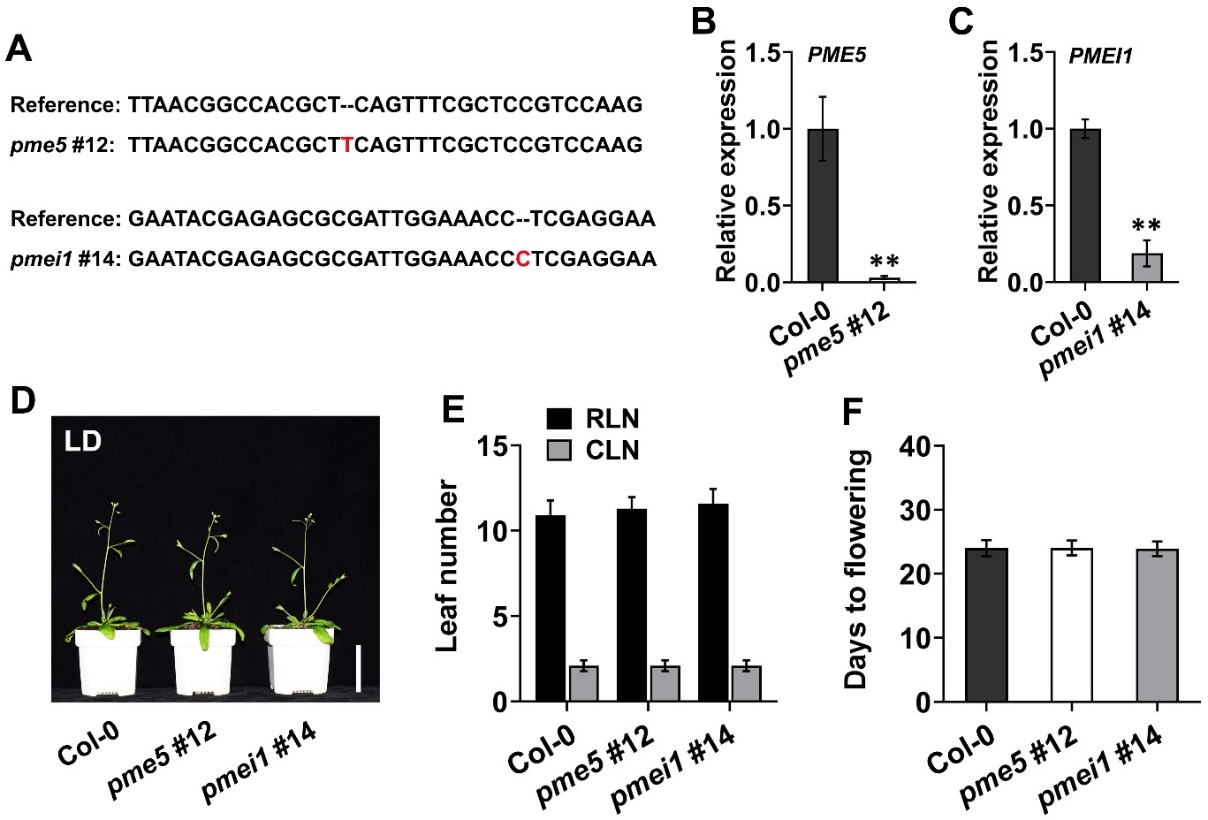


**Figure S4. CRISPR/Cas9 mutants of *PME5* and *PMEI1* do not display flowering phenotype.** (**A**) The mutated sequences of *PME5* and *PMEI1* in *pme5* and *pmei1* plants. *pme5* #12 harbores a 1-bp insertion in the nucleotide at 208 bp of *PME5* coding region, which leads to premature termination and the generation of peptide with 86 amino acids. *pmei1* #14 has a 1-bp insertion in the nucleotide at 190 bp of *PMEI1* coding region, generating a truncated protein of 76 amino acids. (**B** and **C**) RT-qPCR detection of *PME5 and PMEI1* expression levels in *pme5* and *pmei1* seedlings (n = 3. Data are representative of three biological replicates), respectively. *ACT2* was amplified as an internal control. (**D**) 35-day-old Col-0, *pme5* and *pmei1* plants grown in LD conditions. Scale bar = 5 cm. (**E**) Numbers of rosette leaves (RLN) and cauline leaves (CLN) of Col-0, *pme5* and *pmei1* plants in LD (n ≥ 30 plants per genotype. Data are representative of three biological replicates). (**F**) Days to flowering of Col-0, *pme5* and *pmei1* plants in LD (n ≥ 30 plants per genotype. Data are representative of three biological replicates). Error bars represent SD. ***P* < 0.001, Student’s *t*-test.


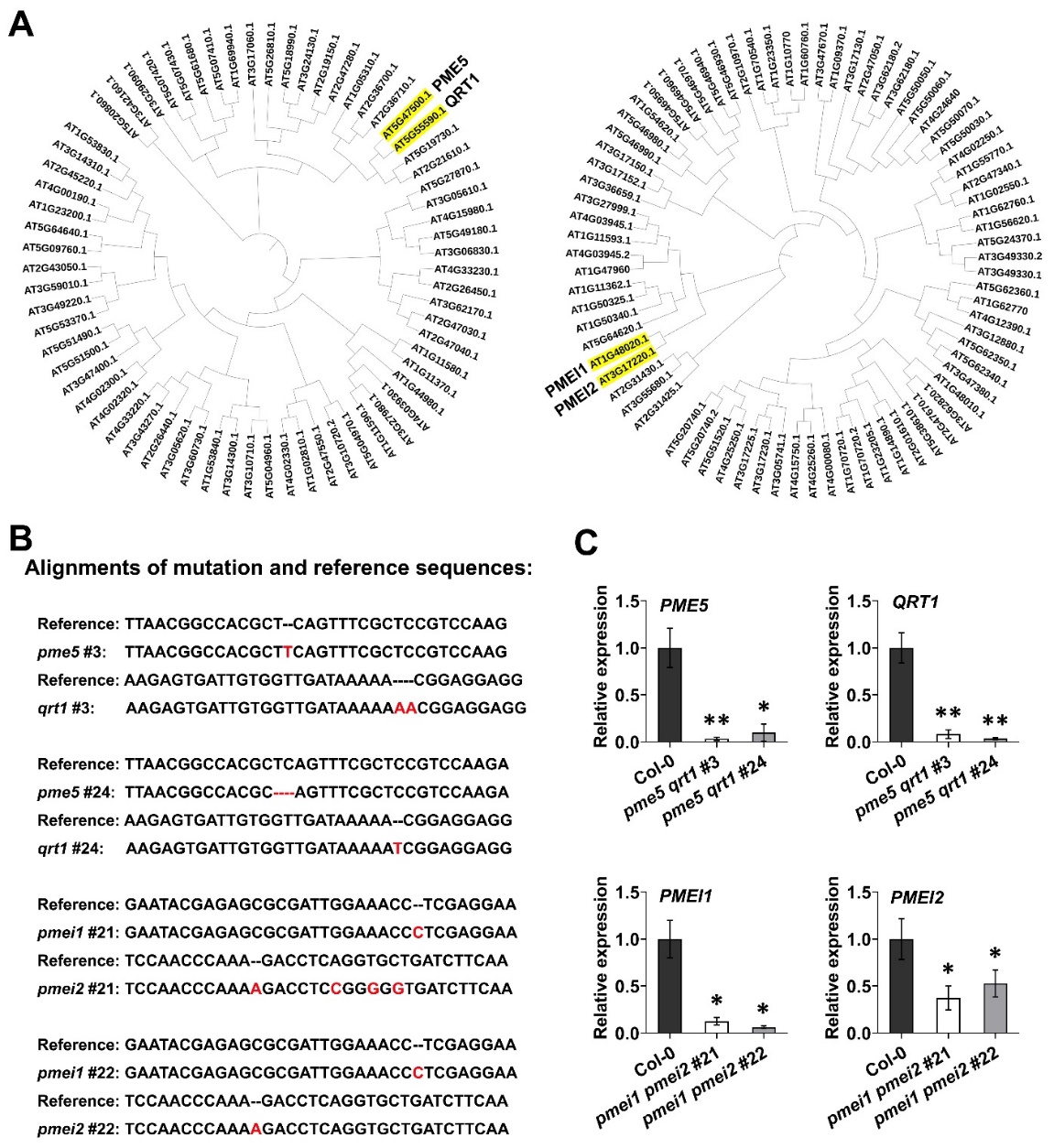


**Figure S5. Phylogenetic analysis, mutated sequences and gene expression levels of double mutant plants.** (**A**) Phylogenetic analysis of PME and PMEI families in Arabidopsis. *PME5* and *QRT1, PMEI1* and *PMEI2* are located in the same clades, respectively*.* (**B**) Mutated sequences in *pme5 qrt1* #3*, pme5 qrt1* #24, *pmei1 pmei2* #21 and *pmei1 pmei2* #22 plants. The mutated nucleotides are highlighted in red color. (**C**) RT-qPCR detection of *PME5*, *QRT1*, *PMEI1* and *PMEI2* expression in corresponding double mutants. *ACT2* was amplified as an internal control (n = 3. Data are representative of three biological replicates). Error bars represent SD. **P* < 0.05, ***P* < 0.001, Student’s *t*-test.


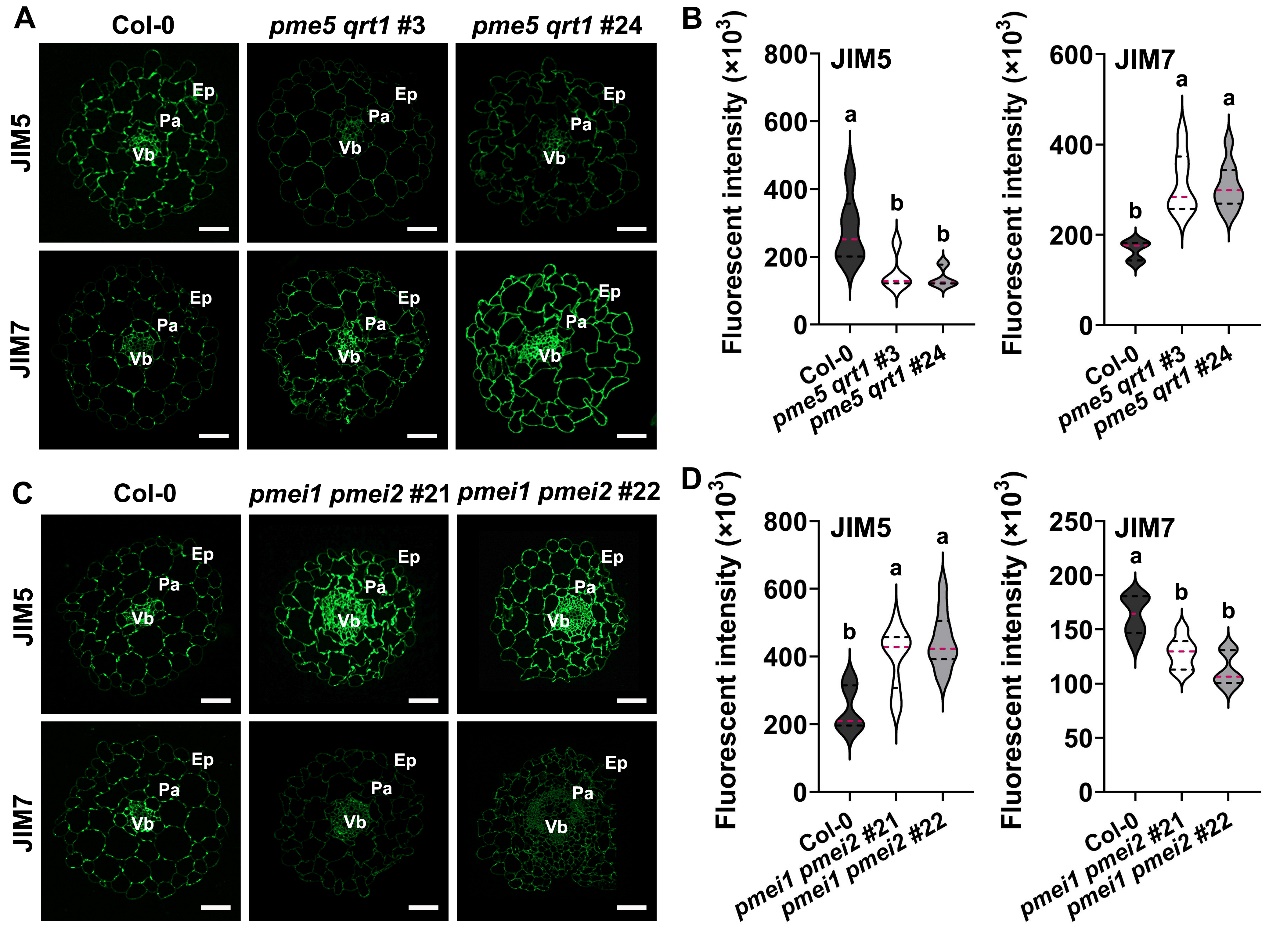


**Figure S6. Immunolabelling of HG methyl-esterification level in double mutants.** (**A**) Immunolabelling on the cross sections of 11-day-old Col-0 and *pme5 qrt1* hypocotyls using JIM5 and JIM7 antibodies. Ep, epidermis; Pa, parenchyma; Vb, vascular bundle. Scale bar = 50 μm. (**B**) Fluorescent intensity of immunolabelling images from cross sections of hypocotyls observed in (A) (n ≥ 6 images from at least 3 seedlings per genotype. Data are representative of three biological replicates). (**C**) Immunolabelling on the cross sections of 11-day-old Col-0 and *pmei1 pmei2* hypocotyls using JIM5 and JIM7 antibodies. Scale bar = 50 μm. (**D**) Fluorescent intensity of immunolabelling images from cross sections of hypocotyls observed in (C) (n ≥ 7 images from at least 3 seedlings per genotype. Data are representative of three biological replicates). Lowercase letters indicate significantly different groups as determined by one-way ANOVA with post-hoc Tukey's test (*P* < 0.05).


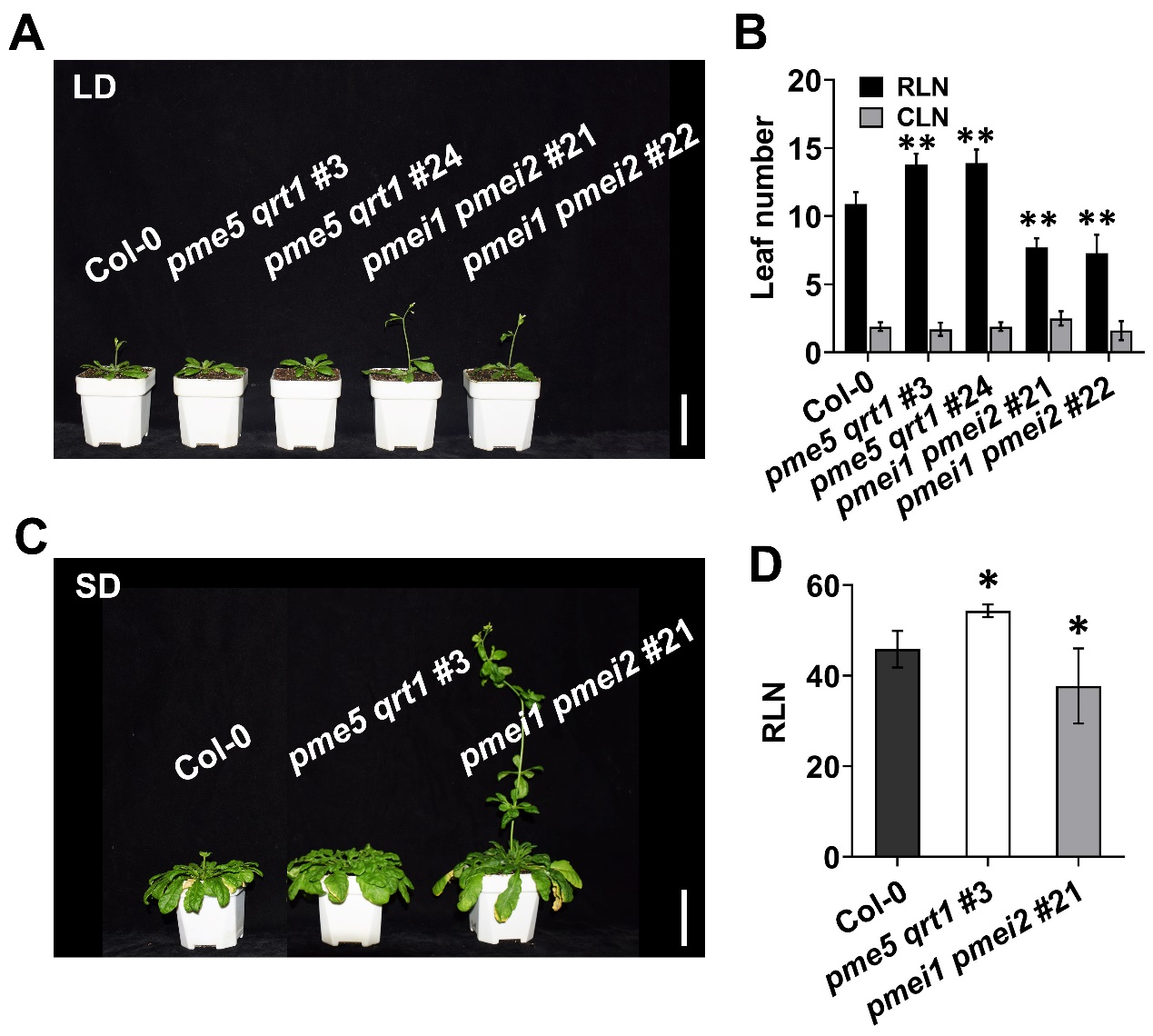


**Figure S7. *pme5 qrt1* and *pmei1 pmei2* mutant plants display late and early-flowering phenotype, respectively.** (**A**) 30-day-old Col-0, *pme5 qrt1*, *pmei1 pmei2* plants grown in LD conditions. Scale bar = 5 cm. (**B**) Numbers of rosette leaves (RLN) and cauline leaves (CLN) of Col-0, *pme5 qrt1* and *pmei1 pmei2* plants in LD condition (n ≥ 30 plants per genotype. Data are representative of three biological replicates). (**C**) Seven-week-old Col-0, *pme5 qrt1* and *pmei1 pmei2* plants in SD conditions. Scale bar = 5 cm. (**D**) Numbers of rosette leaves (RLN) of Col-0, *pme5 qrt1* and *pmei1 pmei2* plants in SD conditions (n ≥ 30 plants per genotype. Data are representative of three biological replicates). Error bars represent SD. **P* < 0.05, ***P* < 0.001, Student’s *t*-test.


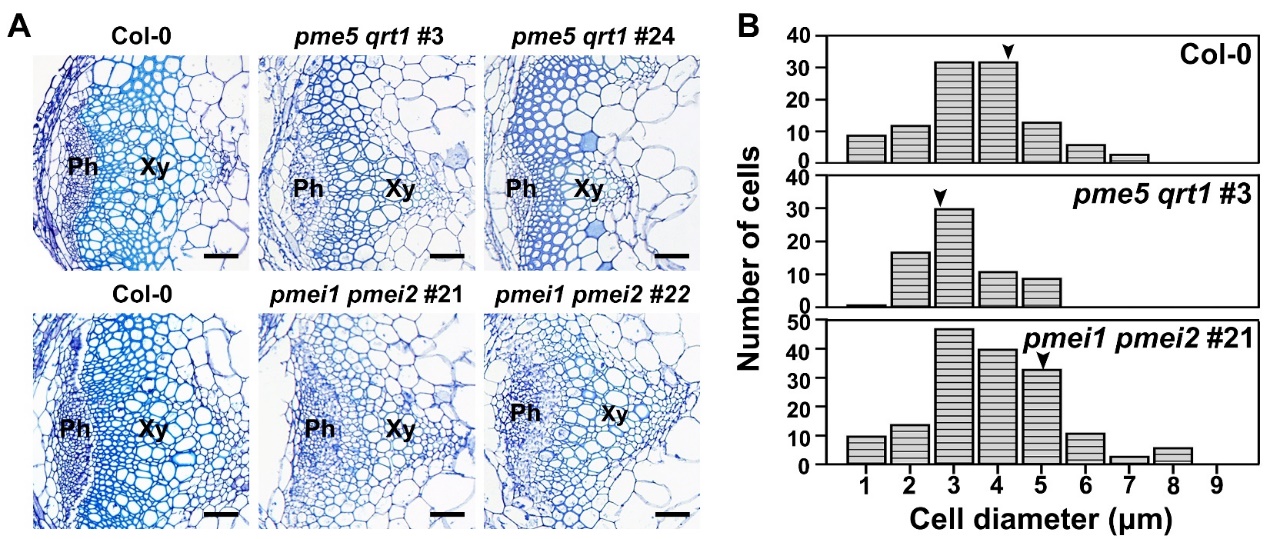


**Figure S8. Phloem development in Col-0, *pme5 qrt1* and *pmei1 pmei2* plants.** (**A**) Transverse sections of basal inflorescence stems in 35-day-old Col-0, *pme5 qrt1* and *pmei1 pmei2* plants. Ph, phloem; Xy, xylem. Scale bar = 50 µm. (**B**) Distribution of the diameter of phloem cells in inflorescence stems of 35-day-old Col-0, *pme5 qrt1* and *pmei1 pmei2* plants (n ≥ 68 per genotype. Data are representative of two biological replicates). Arrowheads show the average diameter of phloem cells.


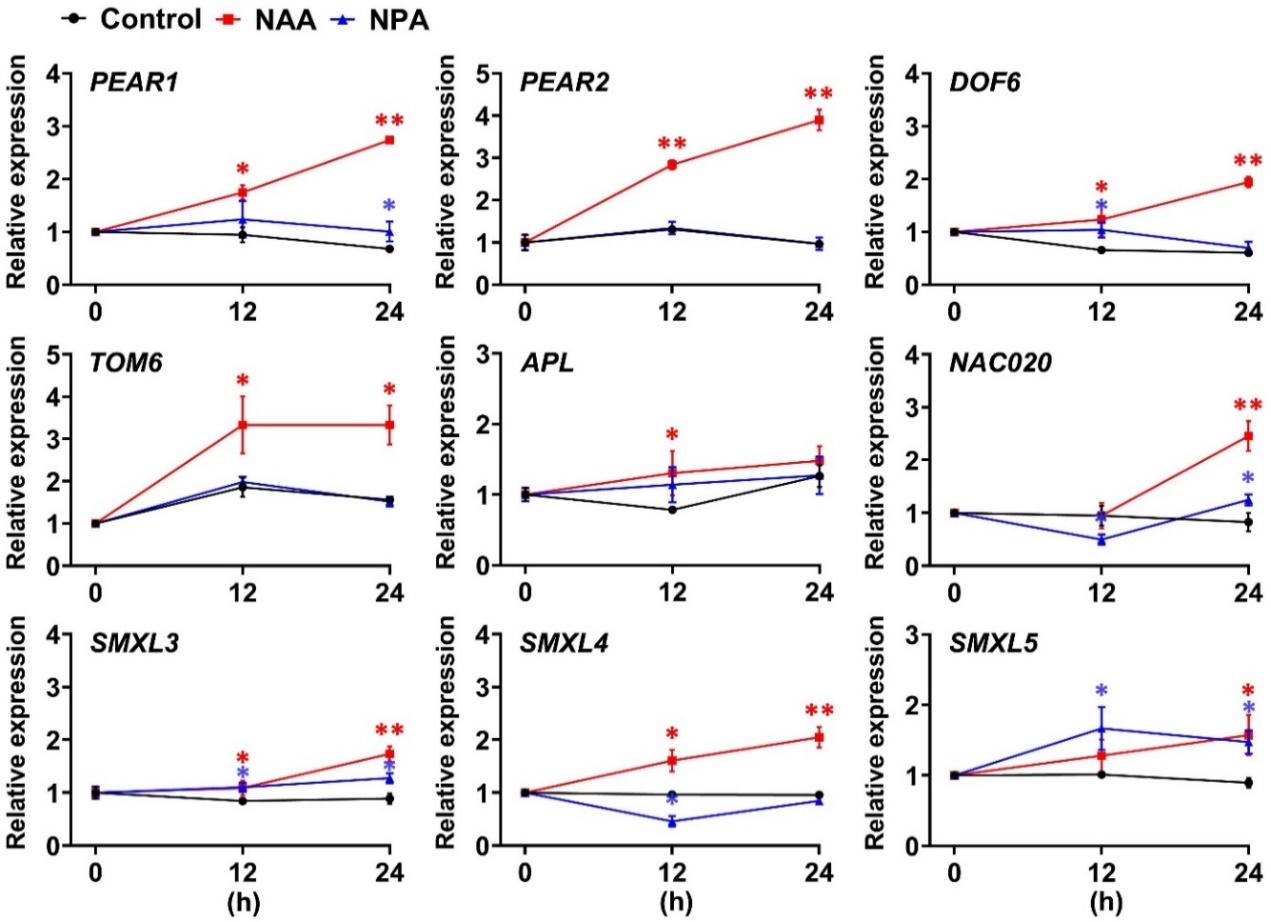


**Figure S9. Relative expression levels of phloem differentiation-related genes upon NAA and NPA treatments.** Gene expression levels were detected by RT-qPCR in 6-day-old Col-0 seedlings grown on ½ MS medium plates with or without 0.5 μM NAA or 20 μM NPA for 12 h or 24 h (n = 3. Data are representative of three biological replicates). *ACT2* was amplified as an internal control. Error bars represent SD. **P* < 0.05, ***P* < 0.001, Student’s *t*-test.

All primer sequences used for gene cloning are listed in Table S1.

| **Accession Number** | **Primer Name** | **Sequence (5'-3')** |
| --- | --- | --- |
| AT5G47500 | PME5 CDS-F | ATGGCGCAACTTACTAATTCC |
|  | PME5 CDS-R | AGCATCTCGAGGAGCGATC |
| AT1G22710 | pSUC2-F | TAAAATCTGGTTTCATATTAATTTCAC |
|  | pSUC2-R | ATTTGACAAACCAAGAAAGT |
| AT1G65480 | FT CDS-F | ATGTCTATAAATATAAGAGACCCTCT |
|  | FT CDS-R | AAGTCTTCTTCCTCCGCAGC |
| AT4G08150 | pKNAT1-F | GATCTAGAGCCCTAGGATTTGA |
|  | pKNAT1-R | ACCCAGATGAGTAAAGATTTGAG |
| AT1G48020 | PMEI1-BsF | ATATATGGTCTCGATTGAGCGCGATTGGAAACCTCGGTT |
|  | PMEI1-F0 | TGAGCGCGATTGGAAACCTCGGTTTTAGAGCTAGAAATAGC |
| AT3G17220 | PMEI2-R0 | AACAAAGACCTCAGGTGCTGATCAATCTCTTAGTCGACTCTAC |
|  | PMEI2-BsR | ATTATTGGTCTCGAAACAAAGACCTCAGGTGCTGATCAA |
| AT5G55590 | QRT1-BsF | ATATATGGTCTCGATTGTTGTGGTTGATAAAAACGGGTT |
|  | QRT1-F0 | TGTTGTGGTTGATAAAAACGGGTTTTAGAGCTAGAAATAGC |
| AT5G47500 | PME5-R0 | AACCGCTCAGTTTCGCTCCGTCCAATCTCTTAGTCGACTCTAC |
|  | PME5-BsR | ATTATTGGTCTCGAAACCGCTCAGTTTCGCTCCGTCCAA |

All primers used for qPCR are listed in Table S2.

| **Accession Number** | **Primer Name** | **Sequence (5'-3')** |
| --- | --- | --- |
| AT3G18780 | ACT2-qF | CTTGCACCAAGCAGCATGAA |
|  | ACT2-qR | CCGATCCAGACACTGTACTTCCTT |
| AT1G48020 | PMEI1-qF | ACACAAGCGAGAGCTACACA |
|  | PMEI1-qR | TCCTCGAGGTTTCCAATCGC |
| AT5G47500 | PME5-qF | CTTTATGCGACGATGCTGGC |
|  | PME5-qR | CGACCCGAATCTTGACGCTA |
| AT5G55590 | C-QRT1-qF | ACTTAGGAAGGGCTTGGGGA |
|  | C-QRT1-qR | ACTCCCCGAACATCACTTTCC |
| AT5G47500 | C-PME5-qF | CGGGTTTTACGGTGCACAAG |
|  | C-PME5-qR | ATGGAGCGGCCATTACCAAA |
| AT1G48020 | C-PMEI1-qF | GAGAGCGCGATTGGAAACCT |
|  | C-PMEI1-qR | TCCATCCAATGCAGCAGAAAC |
| AT3G17220 | C-PMEI2-qF | TCAAGACAGCAACCAACCCC |
|  | C-PMEI2-qR | TGTTCAACCCTTTGCCATCG |
| AT2G37590 | PEAR1-qF | AGCAGCACTGAAATGTCCGA |
|  | PEAR1-qR | CCACGTGTCCAGTATCTCCG |
| AT5G02460 | PEAR2-qF | AATCCGACCAGGTTCGATGG |
|  | PEAR2-qR | AAGTGGCGAGGTTGAGTGAG |
| AT3G45610 | DOF6-qF | TCCCAATCGGTGGTGCTTAC |
|  | DOF6-qR | AACTCGCCGTTGGGAATGAT |
| AT1G49410 | TOM6-qF | AGCCATGTTCCCAGGAATGTT |
|  | TOM6-qR | GGGAGCAGCACGGATAATGA |
| AT1G79430 | APL-qF | CCTCTTAGTTTCCCGCCGTT |
|  | APL-qR | TCTGCAGCATTGTTACCCGT |
| AT1G54330 | NAC020-qF | GAATGGCGCCCATGAGTTTG |
|  | NAC020-qR | TCTCGAGCTCAATGGCTTGG |
| AT3G52490 | SMXL3-qF | CTATGAGAGCTGGAGGCTGC |
|  | SMXL3-qR | GCATGTCCTCTCCGTCTAGC |
| AT4G29920 | SMXL4-qF | CCAAGGGGAGGTATACGAGGA |
|  | SMXL4-qR | AATAAAAGCCGAAGCTAATCAACA |
| AT5G57130 | SMXL5-qF | ACTCCTCTCCATGTTGCAGC |
|  | SMXL5-qR | GTTGAAGACGAGAAGGGGCA |
